# Leveraging Random Effects in Cistrome‐Wide Association Studies for Decoding the Genetic Determinants of Prostate Cancer

**DOI:** 10.1002/advs.202400815

**Published:** 2024-08-05

**Authors:** Mengting Shao, Min Tian, Kaiyang Chen, Hangjin Jiang, Shuting Zhang, Zhenghui Li, Yan Shen, Feng Chen, Baixin Shen, Chen Cao, Ning Gu

**Affiliations:** ^1^ Key Laboratory for Bio‐Electromagnetic Environment and Advanced Medical Theranostics School of Biomedical Engineering and Informatics Nanjing Medical University Nanjing 211166 P. R. China; ^2^ Center for Data Science Zhejiang University Hangzhou 310058 P. R. China; ^3^ Department of Urology The Second Affiliated Hospital of Nanjing Medical University Nanjing 210011 P. R. China; ^4^ Nanjing Key Laboratory for Cardiovascular Information and Health Engineering Medicine Institute of Clinical Medicine Nanjing Drum Tower Hospital Medical School Nanjing University Nanjing 210093 P. R. China

**Keywords:** cistrome‐wide association studies, kernel method, nonlinear effect, prostate cancer

## Abstract

Cistrome‐wide association studies (CWAS) are pivotal for identifying genetic determinants of diseases by correlating genetically regulated cistrome states with phenotypes. Traditional CWAS typically develops a model based on cistrome and genotype data to associate predicted cistrome states with phenotypes. The random effect cistrome‐wide association study (RECWAS), reevaluates the necessity of cistrome state prediction in CWAS. RECWAS utilizes either a linear model or marginal effect for initial feature selection, followed by kernel‐based feature aggregation for association testing is introduced. Through simulations and analysis of prostate cancer data, a thorough evaluation of CWAS and RECWAS is conducted. The results suggest that RECWAS offers improved power compared to traditional CWAS, identifying additional genomic regions associated with prostate cancer. CWAS identified 102 significant regions, while RECWAS found 50 additional significant regions compared to CWAS, many of which are validated. Validation encompassed a range of biological evidence, including risk signals from the GWAS catalog, susceptibility genes from the DisGeNET database, and enhancer‐domain scores. RECWAS consistently demonstrated improved performance over traditional CWAS in identifying genomic regions associated with prostate cancer. These findings demonstrate the benefits of incorporating kernel methods into CWAS and provide new insights for genetic discovery in complex diseases.

## Graphical abstract

The study introduces the random effect cistrome‐wide association study (RECWAS), which improves traditional CWAS by incorporating kernel machine‐based feature aggregation. RECWAS identifies more ChIP‐seq peaks associated with prostate cancer and other complex diseases, providing deeper biological insights. Its ability to capture nonlinear interactions between single nucleotide polymorphisms increases the power of CWAS, as demonstrated by comprehensive simulations and real data analysis.

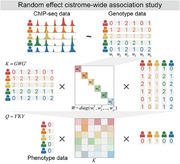

## 1 Introduction

Since its inception in 2005, genome‐wide association studies (GWAS) have emerged as a powerful tool for identifying genetic variants linked to complex human diseases and traits.^[^ ^1^, ^2^ ^]^ GWAS has been instrumental in dissecting the genetic landscape of various diseases, such as mental disorders,^[^ ^3^, ^4^, ^5^ ^]^ cardiovascular disease,^[^ ^6^, ^7^ ^]^ and cancer,^[^ ^8^, ^9^, ^10^ ^]^ leading to the discovery of thousands of associated loci. These findings have significantly advanced our understanding of the genetic underpinnings of these diseases. Despite these advances, GWAS faces challenges,^[^ ^11^ ^]^ particularly in distinguishing causal variants due to linkage disequilibrium (LD).^[^ ^12^ ^]^ Moreover, the predominance of significant signals in non‐coding regions hampers the ability to derive clear biological insights.^[^ ^13^, ^14^ ^]^ Addressing these challenges is vital, necessitating the development of novel algorithms to improve the power of GWAS and deepen our understanding of genetic mechanisms in disease.

Recent years have seen an influx of multi‐omics datasets from extensive population cohorts, encompassing genomics, transcriptomics, and epigenomics, among others.^[^ ^15^, ^16^, ^17^ ^]^ Projects like the Genotype‐Tissue Expression (GTEx)^[^ ^18^ ^]^ have been pivotal, offering gene expression data across as many as 54 tissues from several hundred donors. These datasets, facilitated by advances in high‐throughput sequencing technology, provide invaluable resources for enhancing GWAS. A prime example is transcriptome‐wide association studies (TWAS), which have leveraged gene expression to bridge genetic variants with human diseases, elucidating the genetic mechanisms of complex diseases since their first application in 2015.^[^ ^19^, ^20^ ^]^ TWAS has become a predominant post‐GWAS algorithm, identifying numerous disease susceptibility genes in various complex traits and diseases.^[^ ^21^, ^22^, ^23^, ^24^ ^]^ Traditionally, TWAS employs a regression model that uses gene expression as the outcome and single nucleotide polymorphism (SNP) genotype data as predictors, effectively creating a gene expression imputation model.^[^ ^25^ ^]^ This model is then applied to genotype‐phenotype data to estimate genetically regulated gene expression. However, recent advancements suggest that substituting the linear model in the second step of the traditional TWAS protocol with kernel methods can increase statistical power and reduce type I errors.^[^ ^26^, ^27^ ^]^ Further, the separation of feature selection and aggregation components allows for a more nuanced decoding of the genetic basis of diseases,^[^ ^26^, ^27^, ^28^, ^29^, ^30^ ^]^ and other kernel‐based strategies, have been shown to capture non‐linear SNP interactions, thereby enhancing the identification of disease‐associated genes for TWAS.^[^ ^26^, ^27^, ^30^, ^31^, ^32^ ^]^

Incorporating prior biological knowledge has become a significant trend in enhancing the detection of disease susceptibility genes.^[^ ^32^, ^33^, ^34^ ^]^ Recent TWAS innovations, exemplified by sTF‐TWAS,^[^ ^32^ ^]^ have integrated disease‐specific regulatory elements into gene expression predictions, uncovering novel disease genes. Yet, the biological process through which genetic variants influence gene expression is complex, encompassing factors such as transcription factor binding, enhancer activity, DNA methylation, and chromatin accessibility.^[^ ^35^, ^36^ ^]^ In this context, cistrome‐wide association studies (CWAS) represent a significant advancement.^[^ ^37^ ^]^ Using the cistrome to bridge genetic variants and phenotypes, CWAS aims to unravel the complex interactions between genetic variants and the cistrome state. Building upon and complementing TWAS, CWAS utilizes specific cistrome data – like the androgen receptor (AR) and H3K27 acetylation (H3K27ac) in prostate cancer – to assess the impact of SNP alleles on peak intensity, employing methods like LASSO penalized regression or single SNP models. By correlating cistrome activity with genotype, CWAS calculates peak‐trait associations, as demonstrated in prostate cancer studies, where 74 significant AR peaks and 199 H3K27ac peaks were identified. Crucially, further validation using CRISPR interference showed that suppressing 6 AR binding sites (ARBS), genetically determined, significantly reduced the expression of key prostate cancer risk genes like *TMPRSS2* and *BMPR1B*. Additionally, H3K27ac HiChIP data revealed physical interactions between genes associated with prostate development and oncogenesis and CWAS‐identified ARBS and H3K27ac peaks. These findings underscore CWAS's ability to identify SNPs affecting gene expression through regulatory elements, offering profound insights into the genetic basis of diseases. Nonetheless, CWAS is not without limitations, including the potential for peak‐disease associations to reflect correlation rather than causation, and the inability of linear models to capture non‐linear SNP interactions.

In this study, we present the random effect cistrome‐wide association study (RECWAS) algorithm, innovatively designed to address the limitations of current CWAS approaches, particularly their linear assumptions regarding the contribution of SNPs to cistrome state. Unlike traditional CWAS models that typically consider linear or single‐SNP contributions (as in the TOP1 model of CWAS FUSION), RECWAS utilizes the kernel method to account for non‐linear SNP interactions. This integration significantly improves the accuracy of identifying disease‐associated regions. We rigorously tested RECWAS using both simulated and actual prostate cancer datasets, evaluating its efficacy in pinpointing disease susceptibility regions. The results from these analyses underscore the robust potential of RECWAS, not only in elucidating complex genetic mechanisms but also in uncovering previously unrecognized disease‐associated regions. This has profound implications for our understanding of the genetic basis of prostate cancer and potentially other complex diseases, offering a more nuanced and comprehensive genomic analytical tool.

## 2 Results

### 2.1 Overview of RECWAS

**Figure 1:**
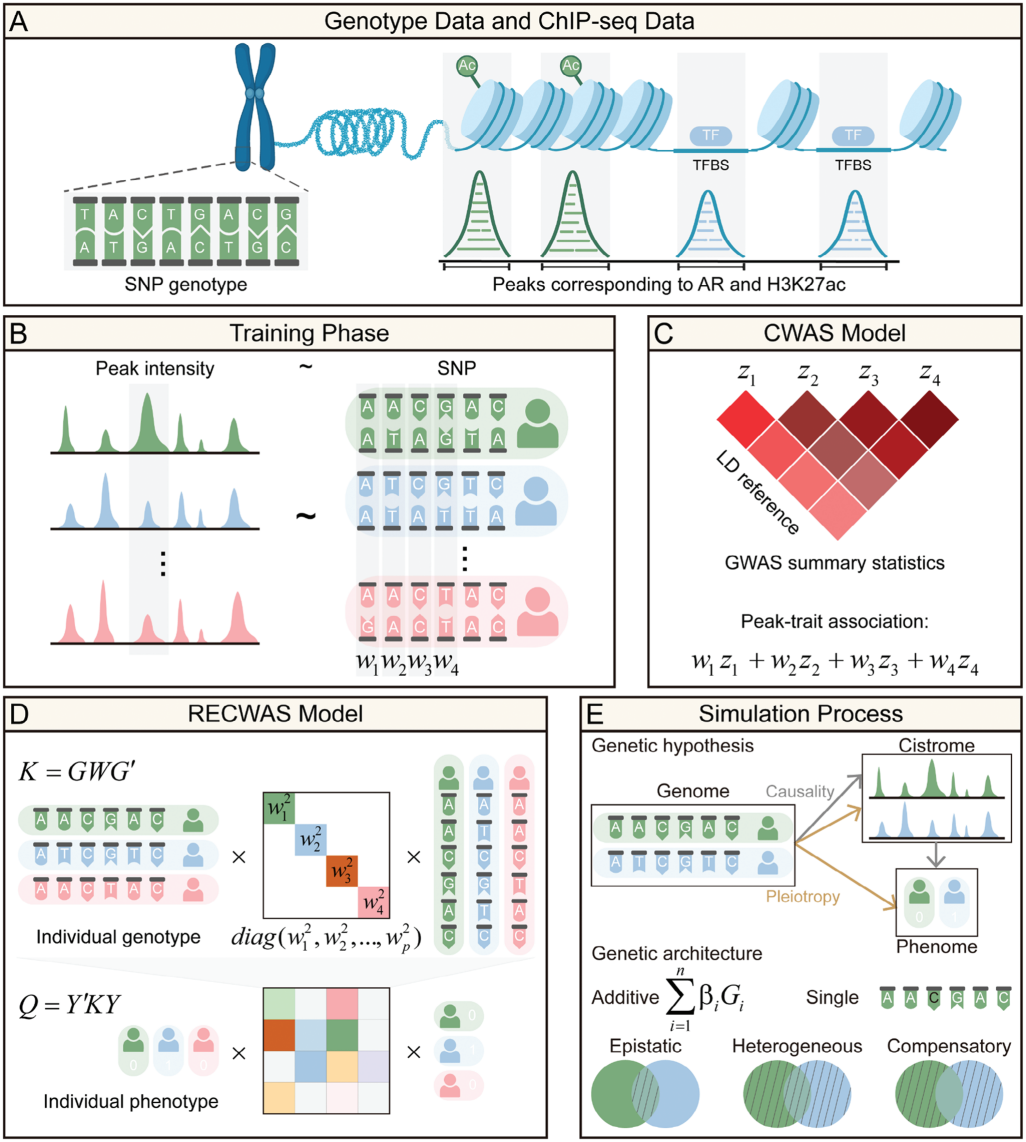
Comparative Overview of the CWAS and RECWAS Models. A) Data Types: Both CWAS and RECWAS models utilize AR binding and H3K27ac peak intensity data from ChIP‐seq, in conjunction with genotype data derived from sequencing. B) Training Phase: This step involves computing the relationship between ChIP‐seq and genotype data, using the LASSO model to compute weights. C) CWAS Model: a linear combination of the calculated weights is used to calculate peak‐trait associations. D) RECWAS Model: In contrast to CWAS, RECWAS employs a SKAT‐like method for assessing associations, focusing on capturing non‐linear effects. E) Simulation Process: The efficacy and robustness of both CWAS and RECWAS models are evaluated through simulations that incorporate various genetic architectures.

We have developed a kernel‐based association approach that connects genetic variants to cistrome activity, utilizing individual genotype and ChIP‐seq data (**Figure** **1**A). This method is comprised of 2 principal components. First, in the training phase (Figure 1B), calculate the weights correlating SNPs with cistrome activity, or using the marginal effect of the SNPs. Second, in contrast to the linear combination model used in traditional CWAS (Figure 1C), RECWAS employs a kernel method for aggregating these weighted variants. This is followed by conducting a score test to assess the association (Figure 1D). To further establish the robustness of our approach, we undertook a series of simulations based on various genetic hypotheses and architectures. These simulations were designed to compare and evaluate the effectiveness of both CWAS and RECWAS (Figure 1E). Comprehensive details of the RECWAS methodology and the simulation procedure are thoroughly presented in the Experimental Section.

In summary, the “random effect” in RECWAS refers to the use of kernel‐based methods to model the combined and potentially non‐linear effects of multiple SNPs on cistrome states, improving the study's ability to identify significant genetic associations compared to traditional CWAS. Traditional CWAS generally utilize fixed effects models, such as linear regression, where each SNP's effect on the cistrome state is modeled independently. This approach assumes a linear relationship between genetic variants and phenotypes. The linear models in CWAS are limited in their ability to capture complex, non‐linear interactions among SNPs, potentially leading to an incomplete understanding of genetic contributions to cistrome states. In contrast, RECWAS employs random effects through kernel methods (e.g., sequence kernel association test (SKAT)), which allow for the aggregation of multiple genetic variants' effects within a genomic region. These methods model the combined influence of SNPs on the cistrome state, capturing both linear and non‐linear interactions. By using a kernel‐based approach, RECWAS improves the detection of associations between genetic variants and cistrome states. This method can identify regions where multiple SNPs collectively contribute to cistrome variation, leading to better identification of disease‐associated genomic regions.

### 2.2 Comparative Performance of RECWAS and CWAS in Simulated Genetic Scenarios

Type I errors refer to the probability of falsely rejecting a true null hypothesis. Controlling type I error is crucial in GWAS to ensure the validity and reliability of the findings. Our null simulations revealed that the type I error rate of RECWAS, defined by a 5% cut‐off (established through simulating traits under the null distribution), is 0.0501. This value aligns closely with the targeted type I error rate of α = 0.05. Similarly, traditional CWAS maintains a well‐controlled 5% cut‐off at 0.0513. These findings show that the type I error of RECWAS is effectively managed. Additionally, it's noteworthy that other kernel‐based wide association studies, such as kTWAS^[^ ^27^ ^]^ and VC‐TWAS^[^ ^26^ ^]^ have previously demonstrated well‐controlled type I error rates, further supporting the reliability of RECWAS.

**Figure 2:**
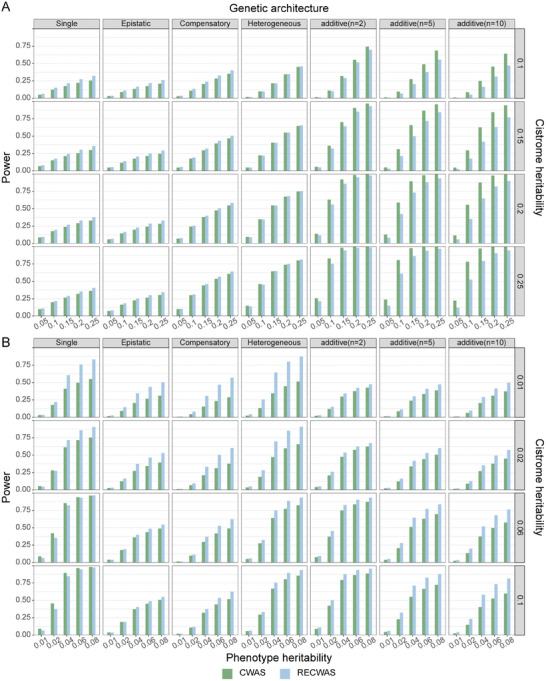
Assessment of the statistical power of CWAS and RECWAS models across different genetic architectures and heritability levels. This figure illustrates the comparative effectiveness of CWAS and RECWAS under different genetic scenarios: A) causality and B) pleiotropy. The x‐axis represents the heritability of the phenotype, while the left y‐axis measures the statistical power of the models and the right y‐axis denotes the cistrome heritability. This dual‐axis representation allows for a comprehensive evaluation of how CWAS and RECWAS perform concerning varying levels of phenotype and cistrome heritability under different genetic conditions.

In simulations assessing statistical power, RECWAS consistently demonstrates superior performance over CWAS in most scenarios, primarily attributed to its ability to capture non‐linear SNP interactions (**Figure** **2**). This comparative analysis was conducted under both causality and pleiotropy models. In these simulations, RECWAS significantly outperformed CWAS in a majority of the scenarios. Specifically, under the causality model, RECWAS showed improved power in non‐additive scenarios compared to CWAS, while CWAS exhibited greater power in additive scenarios (Figure 2A). In the context of the pleiotropy model, RECWAS surpassed CWAS in both additive and non‐additive scenarios, with this advantage becoming more pronounced in cases of elevated heritability (Figure 2B). Moreover, when considering scenarios where a single SNP is presumed to determine the cistrome state, RECWAS displayed markedly better performance than CWAS, particularly notable in situations with high cistrome heritability. In conclusion, these simulation results demonstrate RECWAS's superior ability to capture complex SNP interactions, marking it as an effective tool in CWAS analysis, particularly in scenarios involving non‐linear effects and low cistrome heritability for the pleiotropy model. The full evaluation results of the simulation are illustrated in Figures S1 and S2 (Supporting Information).

Furthermore, we evaluate the performance of RECWAS using various kernels in simulation data, including the linear kernel, weighted quadratic kernel, 2wayIX kernel, identity by state (IBS) kernel, and weighted IBS kernel. These evaluations are conducted under different genetic scenarios such as causality and pleiotropy, with both additive and non‐additive models. Simulation results indicate that under the causality scenario, the weighted linear kernel (RECWAS) performs best with the additive model and demonstrates comparable power with the best kernel (IBS kernel) in nonadditive models. Under the pleiotropy scenario, the IBS kernel shows the best performance in additive models, while RECWAS exhibits comparable power. As heritability increases, the power of RECWAS increases significantly. In nonadditive models, RECWAS achieves the highest power in the “Single” model and demonstrates comparable power to the IBS kernel in other non‐additive models. The full evaluation results of the simulation are illustrated in Figures S3 and S4 (Supporting Information).

### 2.3 Enhanced Detection of Associations by RECWAS in Prostate Cancer Analysis

#### 2.3.1 Superior Identification of Significant Peaks with RECWAS

**Figure 3:**
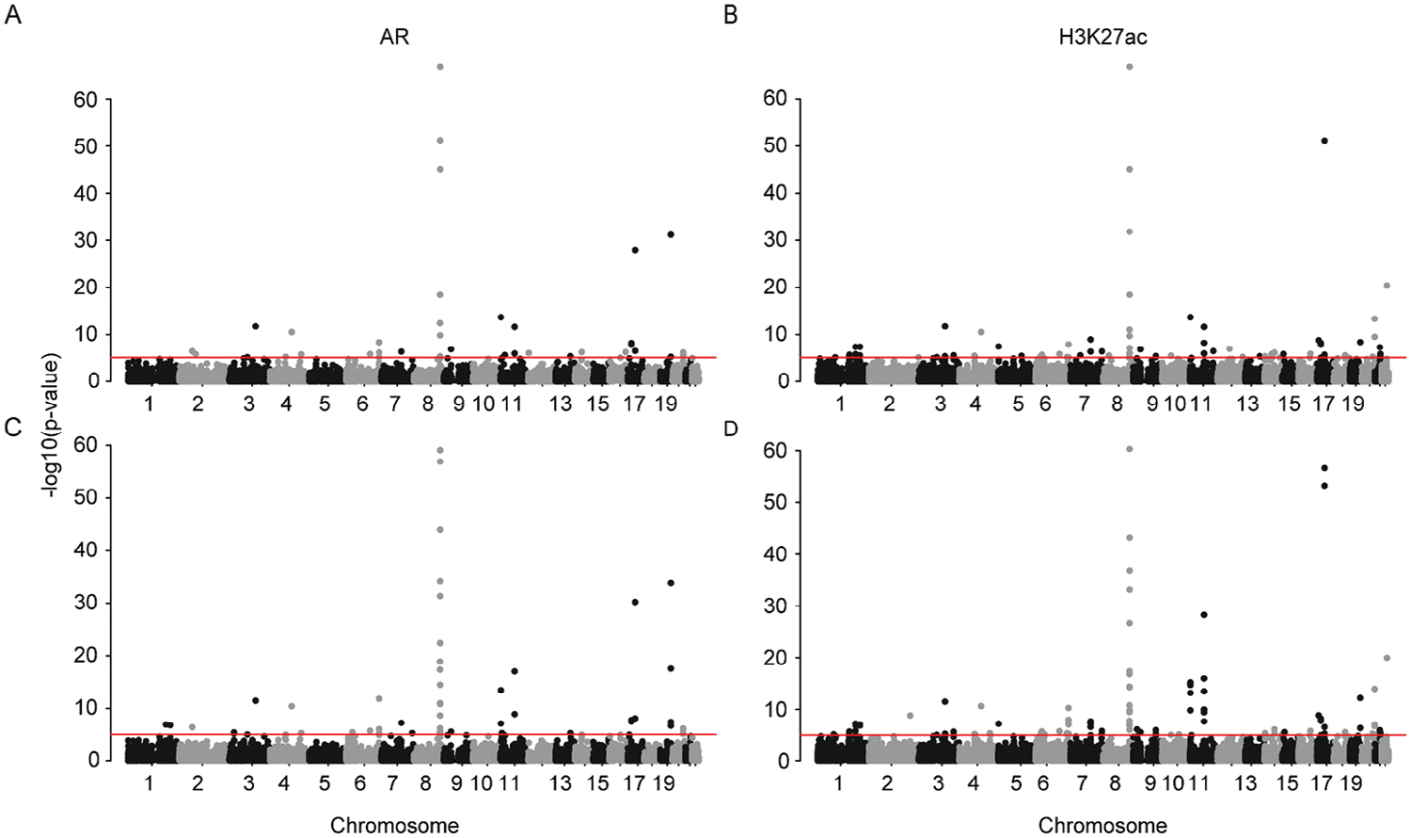
Comparative analysis of CWAS and RECWAS in prostate cancer. A) AR peak associations identified by CWAS. B) H3K27ac peak associations identified by CWAS. C) AR peak associations identified by RECWAS. D) H3K27ac peak associations identified by RECWAS.

**Figure 4:**
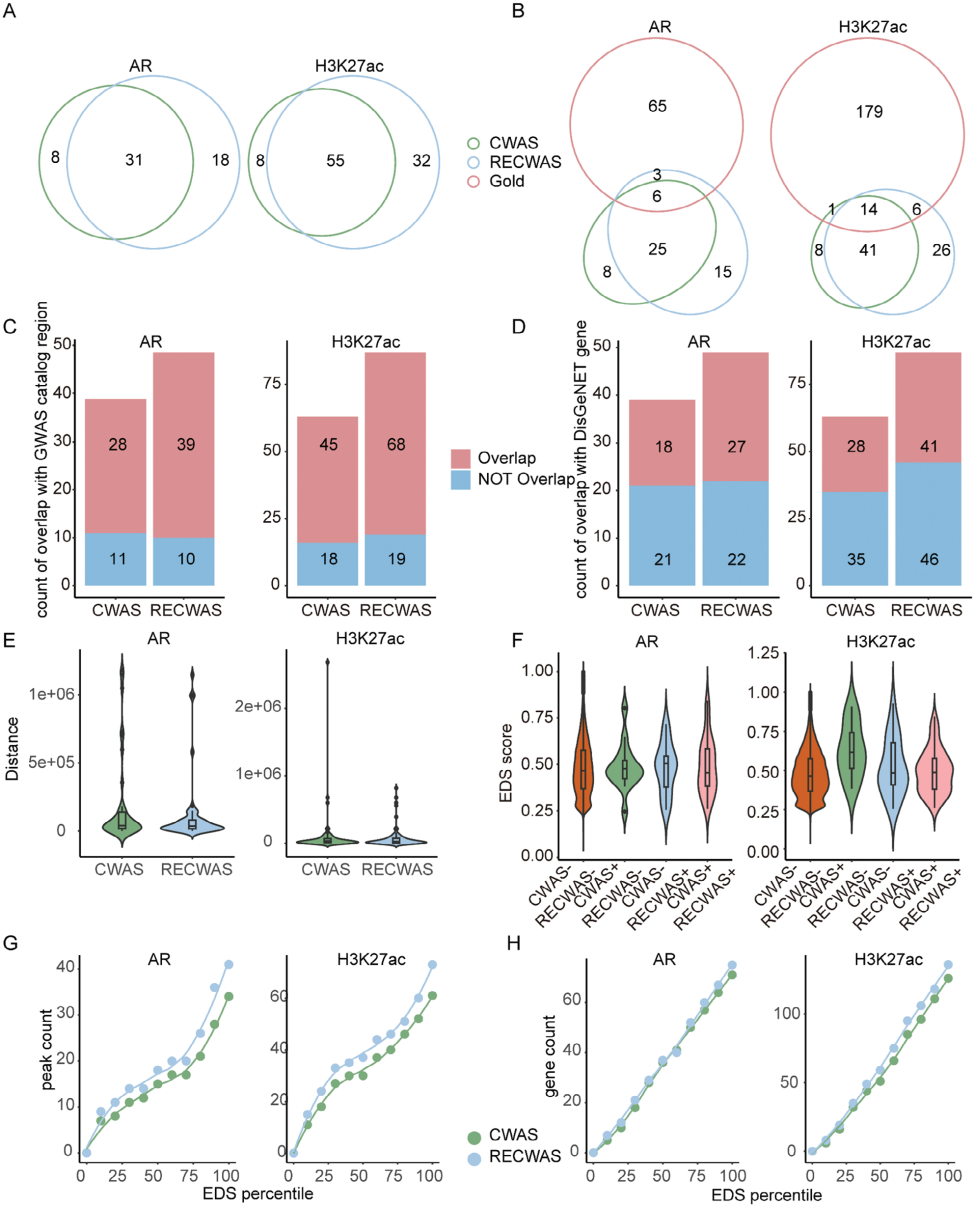
Comparative validation analysis of significant peaks identified by CWAS and RECWAS. A) Overlap of significant peaks identified by CWAS and RECWAS. B) Overlap of gold peak datasets with significant results of CWAS and RECWAS. C) The proportion of significant peaks located in prostate cancer GWAS risk region. D) The proportion of significant peaks located in prostate cancer susceptibility regions. E) Distance distribution between significant peaks and prostate cancer susceptibility genes. F) EDS distribution of different peak groups. G) Count distribution of significant peaks located in genes with EDS percentiles. H) Count distribution of genes within significant peaks located in.

In the application of CWAS and RECWAS to prostate cancer datasets, Manhattan plots (**Figure** **3**) delineate the comparative efficacy of these models. CWAS identified 39 AR and 63 H3K27ac significant peaks (Figure 3A,B), whereas RECWAS demonstrated an increased detection capacity, identifying 49 AR and 87 H3K27ac significant peaks (Figure 3C,D), using the same established *p* value thresholds of <0.05/5580 for AR and <0.05/17199 for H3K27ac. Importantly, RECWAS detected 18 novel AR peaks and 32 novel H3K27ac peaks that were not detected by CWAS, indicating its superior sensitivity in identifying potential disease‐associated regions. Among the 87 significant H3K27ac peaks identified by RECWAS, 55 were also recognized by CWAS (**Figure** **4**A). Further analysis using prostate cancer gold peak datasets (detailed in the Experimental Section) found that RECWAS identified 3 novel AR and 6 H3K27ac gold peaks, in contrast to CWAS which identified only one H3K27ac gold peak within these datasets (Figure 4B).

#### 2.3.2 RECWAS Identifies More Prostate Cancer‐Related Peaks than CWAS

RECWAS demonstrated a more proficient detection of peak‐trait associations pertinent to prostate cancer risk than CWAS, as evidenced by several biological evidence.

First, RECWAS identified a greater number and proportion of peaks in both the prostate cancer GWAS risk region and the susceptibility‐related region, compared to CWAS. Specifically, CWAS identified 39 significant AR peaks and 63 H3K27ac peaks, with 72% of AR peaks and 71% of H3K27ac peaks located in the GWAS risk region. In contrast, significant peaks were identified by RECWAS with 80% of AR peaks and 78% of H3K27ac peaks located in the GWAS risk region. Regarding the prostate cancer susceptibility‐related region, sourced from the DisGeNET^[^ ^38^ ^]^ database, there are 46% of AR peaks and 44% of H3K27ac peaks identified by CWAS located in, whereas RECWAS identified a higher proportion: 55% of AR peaks and 47% of H3K27ac peaks. These findings, illustrated in Figure 4C,D, underscore RECWAS's improved ability to detect more disease susceptibility peaks, potentially uncovering peaks that might be overlooked by CWAS.

Second, our analysis revealed that significant peaks identified by RECWAS were situated closer to prostate cancer susceptibility genes compared to those identified by CWAS. This was quantitatively evidenced by the mean distances: the mean distance for AR peaks in CWAS was 186 893 bp, whereas it was significantly reduced to 119 844 bp in RECWAS. A similar trend was observed for H3K27ac peaks, the mean distance for CWAS peaks was 106 954 bp, which decreased to 77 259 bp in RECWAS (Figure 4E).

Thirdly, extending our analysis to consider the relationship between identified peaks and enhancer domain scores (EDS),^[^ ^39^ ^]^ we drew upon previous studies that have shown a correlation between genes with high EDS and the proximity of CWAS peaks.^[^ ^37^ ^]^ Our findings indicate that peaks uniquely identified by RECWAS tend to cluster more frequently around genes with higher EDS scores, particularly in the AR model. This is evident in the analysis of the ±100 kb genomic region surrounding the centers of genes, where RECWAS displayed an increased concentration of peaks near genes with high EDS. Furthermore, RECWAS identified a greater number of high‐EDS genes close to significant peaks. These detailed observations are meticulously illustrated and explained in Figure 4F,G.

In conclusion, RECWAS identified 50 unique significant peaks, including 18 AR peaks and 32 H3K27ac peaks, while CWAS identified 16 unique significant peaks, comprising 8 AR peaks and 8 H3K27ac peaks. Of the RECWAS‐identified peaks, 10 out of 18 AR peaks and 16 out of 32 H3K27ac peaks mapped to prostate cancer susceptibility genes in the DisGeNET database. In comparison, CWAS‐identified peaks included 1 out of 8 AR peaks and 3 out of 8 H3K27ac peaks mapping to these genes. Additionally, 13 out of 18 AR peaks and 26 out of 32 H3K27ac peaks from RECWAS were located in prostate cancer risk regions listed in the GWAS catalog, compared to 2 out of 8 AR peaks and 5 out of 8 H3K27ac peaks from CWAS. Notably, RECWAS identified 3 out of 18 AR peaks and 6 out of 32 H3K27ac peaks in the prostate cancer gold peak datasets, whereas CWAS identified none. The significant overlap of RECWAS‐identified peaks with known prostate cancer susceptibility genes, risk regions, and gold peak datasets underscores its robustness. Detailed information about the significant peaks identified by both CWAS and RECWAS in prostate cancer is systematically presented in Tables S1 and S2 (Supporting Information) to provide a comprehensive overview and facilitate further investigation.

#### 2.3.3 RECWAS Identifies New Genomic Regions and AR Peaks Crucial for Prostate Cancer

AR in prostate cancer can drive the transcriptional repression of multiple genes, thereby promoting cancer development and progression.^[^ ^40^, ^41^ ^]^ H3K27ac is an epigenetic modification of the histone protein H3, serving as a valuable marker for identifying functional genomic elements across various cell types and conditions. This modification is associated with increased transcriptional activation and is thus defined as an active enhancer mark.^[^ ^42^ ^]^ Although H3K27ac is not specific to prostate cancer or cancer diagnosis, its role in marking active regulatory regions is crucial for understanding gene regulation in various diseases,^[^ ^43^, ^44^, ^45^ ^]^ including cancer.^[^ ^46^ ^]^ Therefore, deeper research into these epigenetic modifications can help uncover the genetic determinants of prostate cancer.

**Figure 5:**
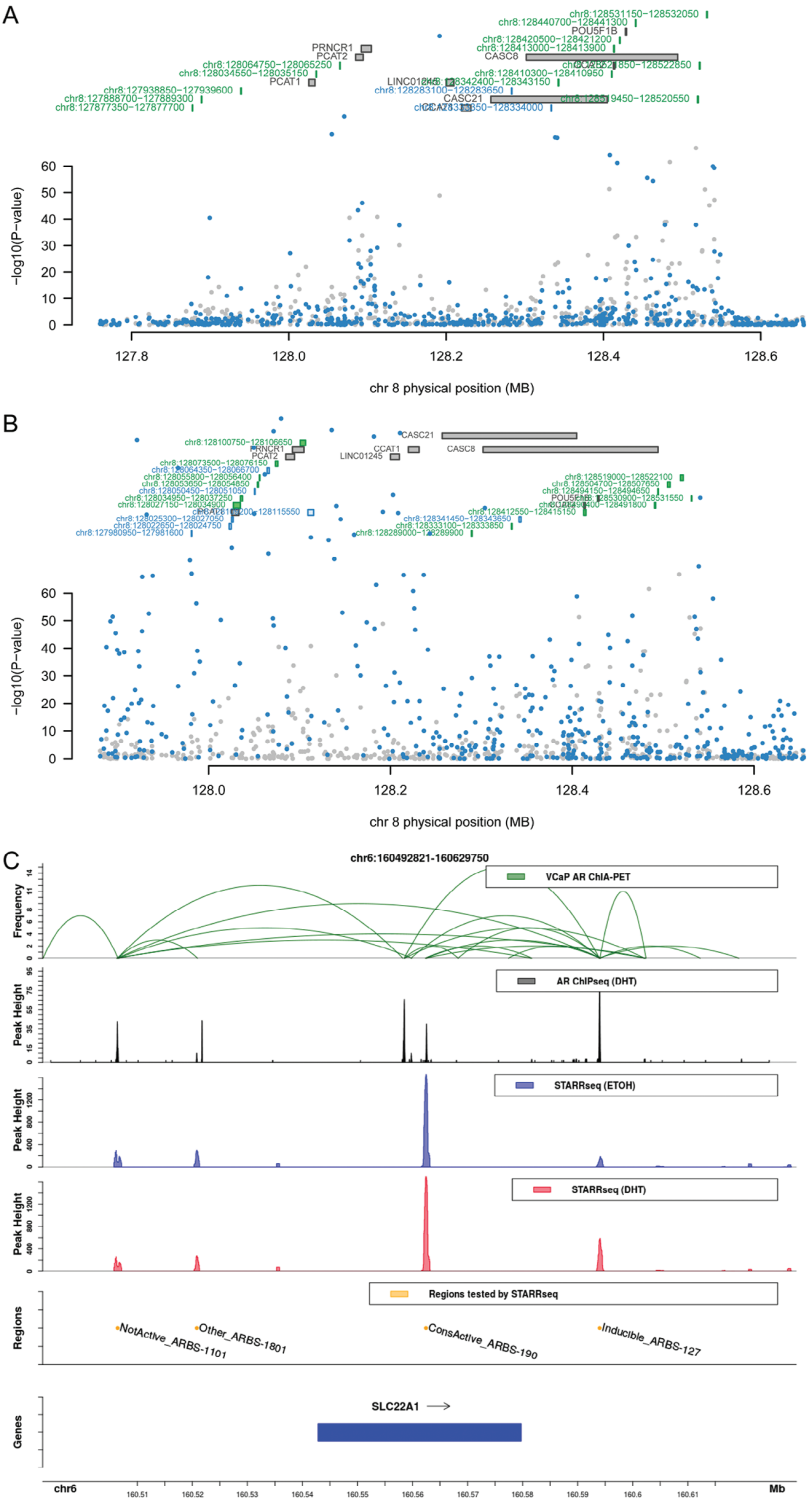
Detailed visualization of RECWAS‐identified peaks in the 8q24 region and AR peak activity. A) 8q24 region in AR model of RECWAS. B) 8q24 region in H3K27ac model of RECWAS. C) AR peak ‘chr6:160561950‐160562800′ (ConsActive_ARBS‐190) activity around SLC22A1 gene download from https://lacklab.shinyapps.io/LSSHL/.^[^ ^53^ ^]^

RECWAS has made progress in identifying novel genomic regions associated with prostate cancer. Specifically, RECWAS identified 18 new AR peaks and 32 H3K27ac peaks. A noteworthy discovery is in the 8q24 genomic region, known for harboring multiple prostate cancer risk variants.^[^ ^47^, ^48^, ^49^ ^]^ In this region, RECWAS uniquely identified 7 AR and 13 H3K27ac peaks near the *CASC8 (cancer susceptibility candidate 8)* and *PCAT1 (prostate cancer‐associated transcript 1)* genes, outperforming the discoveries made by CWAS (refer to Tables S1 and S2 (Supporting Information), and **Figure** **5**A,B for details). The *CASC8* gene has been implicated in several cancers, including prostate cancer.^[^ ^50^ ^]^ Variants in the *CASC8* gene could potentially affect transcription factor binding, influencing prostate cancer risk.^[^ ^51^ ^]^ RECWAS identified 4 novel peaks near *CASC8* that could represent regulatory elements associated with prostate cancer risk. *PCAT1* is another long non‐coding RNA associated with prostate cancer risk. It has been shown to promote prostate cancer cell proliferation.^[^ ^52^ ^]^ RECWAS identified 3 novel peaks near *PCAT1* that may pinpoint enhancer regions driving its tumorigenesis in prostate cancer. Insights from previous functional studies on these genes highlight their importance in cancer biology. The novel peaks identified by RECWAS suggest that these regions may play crucial roles in regulating gene expression and contributing to prostate cancer risk.

These RECWAS‐identified peaks find support in multiple literary sources. For example, the AR peak ‘chr6:160561950‐160562800′, with a RECWAS *p* value of 6.20e‐06 and a CWAS *p* value of 1.73e‐02, has been previously identified in the work of Huang et al.,^[^ ^53^ ^]^ as a prostate cancer‐associated ARBS near the *SLC22A1* gene. This site, known as ConsActive_ARBS‐190, can be verified at the online resource (https://lacklab.shinyapps.io/LSSHL/), with a query for the SLC22A1 gene, as illustrated in Figure 5C. Such cross‐referencing with existing literature not only validates the novel peaks identified by RECWAS but also reinforces their potential significance in prostate cancer research.

## 3 Discussion

GWAS and TWAS have become essential for identifying genes and genomic regions associated with diseases. In this context, Baca et al.^[^ ^37^ ^]^ made a significant contribution by developing CWAS, utilizing ChIP‐seq data to analyze ARBS and regulatory elements linked to prostate cancer, thereby enriching our understanding of its genetic basis. Building on this, we introduce the RECWAS, an innovative algorithm that integrates aggregating features with a SKAT‐like model. RECWAS is designed to discover genomic regulatory regions implicated in diseases, advancing the capabilities of genetic analysis. Our comprehensive simulation studies and real‐data applications in prostate cancer research demonstrate RECWAS's improved performance. It exhibits higher power in the majority of scenarios for both pleiotropy and causality models. This aligns with previous studies that have shown the efficacy of kernel methods in TWAS, particularly in capturing the non‐linear interactions of multiple SNPs.^[^ ^26^, ^27^, ^29^, ^30^, ^31^ ^]^ RECWAS contribute to advancement in genetic research, offering a more nuanced approach to understanding the genetic underpinnings of complex diseases.

In prostate cancer analysis, RECWAS notably outperforms CWAS by identifying a greater number and proportion of significant genomic regions. This improved detection capability extends to key areas such as the prostate cancer GWAS risk region, the susceptibility‐related region, and regions adjacent to genes with high EDS. Crucially, the unique peaks identified by RECWAS are substantiated by existing literature, providing a biologically relevant understanding of their association with prostate cancer risk. These results, supported by diverse biological evidence, not only validate the robustness of RECWAS but also underscore its practical utility in advancing disease research, demonstrating its potential as a useful tool for future genomic studies. To further validate the applicability of RECWAS to nonprostate cancer datasets, we applied RECWAS using pre‐trained weights from whole blood tissue to 4 dbGaP datasets (Table S3, Supporting Information). The results demonstrate that RECWAS can identify peaks that may be missed by CWAS (Figure S5, Supporting Information). Additionally, in the validation using GWAS risk regions extracted from the GWAS catalog and susceptibility genes extracted from the DisGeNET database, the peaks uniquely identified by RECWAS showed a higher number of hits compared to those identified by CWAS. These results demonstrate the broader applicability of RECWAS in identifying significant genetic peaks across various complex diseases.

The current implementation of RECWAS has certain limitations, primarily due to its reliance on individual genotype and phenotype data as input. This dependency can restrict the applicability of RECWAS in studies without readily available individual‐level data. Additionally, the effectiveness of RECWAS is influenced by the sample size; increasing the number of samples has been observed to increase statistical efficacy. Future improvement of RECWAS will focus on accommodating summary‐level statistical data to address these limitations, thereby expanding its applicability and ease of use.

Another limitation is the reliance on pre‐trained weights for specific tissues. Effective analysis requires pre‐trained weights from population‐scale epigenome reference panels relevant to the specific tissue of interest, which poses a challenge in the absence of appropriate datasets. Additionally, the associations identified by RECWAS may correlate with risk without necessarily mediating it. As more epigenome reference panels become available, the utility and accuracy of RECWAS will improve. Further exploration of RECWAS will also focus on the integration of causal inference methods, such as Mendelian randomization, with RECWAS to improve the identification of causal risk cistrome peaks.

Additionally, while RECWAS currently considers SNPs within a 25 kb region, it is recognized that SNPs beyond this range can also influence chromatin states. The inclusion of trans‐cQTLs in future iterations is anticipated to refine the identification of disease‐associated regions. Moreover, considering the broad impact of sex chromosomes on autosomal expression, it has been reported that integrating SNP effects from sex chromosomes presents another avenue for enhancing the efficacy of RECWAS.^[^ ^54^ ^]^ Such integration could not only improve the power of CWAS but also deepen our understanding of the genetic influences on chromatin state dynamics.

There is a growing trend of integrating multiple analytical approaches, such as GWAS, TWAS, and proteome‐wide association study. These integrated approaches have been shown to identify significant clinical risk predictors,^[^ ^55^ ^]^ explore shared molecular pathways between different traits,^[^ ^56^ ^]^ and prioritize disease causal genes.^[^ ^57^ ^]^ In our future work, we are considering the incorporation of RECWAS alongside TWAS. RECWAS identifies regulatory elements and potential causal variants by leveraging chromatin accessibility data. Combining RECWAS with TWAS can improve the identification of functional regulatory elements and genes associated with disease risk, providing a more comprehensive understanding of the genetic architecture underlying complex traits and diseases.

These planned developments reflect our commitment to evolving RECWAS into a more comprehensive and powerful tool in genomic research, capable of addressing the complexities of genetic influences in human diseases.

## 4 Experimental Section

### CWAS Model

The CWAS and RECWAS models use genotype data as input and integrate ChIP‐seq data for AR transcription factor binding sites and H3K27ac histone modifications. This approach positions these cistrome states as a “middle” phenotype, effectively capturing the influence of genetic variants on cistrome states during transcriptional regulation (Figure 1A). CWAS uniquely considers 3 models to comprehensively describe the genetic structure of cistrome activity.

First, the cQTL model takes into account the overall cistrome activity and genotype in a linear relationship, similar to TWAS. It can be defined as

(1) $$Y_{total}∼X_{total}+\varepsilon$$

where *Y_total_* represents the overall cistrome activity for each individual, and *X_total_* is the sum of the maternal haplotypes *H_m_* and paternal haplotypes *H_p_*. Second, the allelic imbalance model replaces total activity with allelic activity, defined as

(2) $$Y_{allelic}∼X_{allelic}+\varepsilon$$

Here, *Y_allelic_* is determined as a log(*N_p_*/*N_m_*), with *N_p_*/*N_m_* representing the number of reads mapping to heterozygous variants of the paternal/maternal haplotype, and *X_allelic_* is the difference of the paternal haplotypes *H_p_* and maternal haplotypes *H_m_*. Lastly, the combined model is denoted as

(3) $$\left[\begin{array}{c} {\tilde{Y}}_{total}\\ {\tilde{Y}}_{allelic} \end{array}\right]∼\left[\begin{array}{c} {\tilde{X}}_{total}\\ {\tilde{X}}_{allelic} \end{array}\right]+\varepsilon$$

This model integrates both the total and allelic activity components. To train the weights *W* between variants and chromatin activities, LASSO and TOP1 models are adopted for each of the aforementioned models (Figure 1B).

The process of calculating associations between the GWAS signal and predicted cistrome activity was carried out using the FUSION software (http://gusevlab.org/projects/fusion/). For each AR or H3K27ac peak, the model with the most significant cross‐validation *p* value was chosen. The genotypes of cis‐SNPs within 50 kb were considered for testing using the equation

(4) $$Z_{peak,trait}=WZ_{snp,trait}$$

Here, *W* is the weight matrix, which is a matrix product between the peak–SNP covariance matrix and the SNP–SNP covariance matrix, and *Z*_*snp*,*trait*_ is the *Z* score vector derived from SNP‐trait association (GWAS summary statistics). Ultimately, these associations were evaluated against a stringent significance threshold of *p* value < 0.05/5580 for AR and *p* value <  0.05/17199 for H3K27ac (Figure 1C).

### RECWAS Model

Prior studies^[^ ^26^, ^27^, ^30^ ^]^ have highlighted the increased statistical power achieved through kernel methods in TWAS, our study adopts the SKAT^[^ ^58^ ^]^ as the chosen kernel method for peak‐trait association analysis. SKAT is particularly adept at leveraging variance‐component score statistics by assessing the cumulative effect of multiple genetic variants within a specific genomic region on a phenotype. To delineate its methodology, the SKAT model for each genomic region *i*, containing *p* variants, establishes a preliminary linear relationship. This relationship is between the genetic variants of the region (denoted as *G_i_*) and the phenotype of interest (*Y_i_*)

(5) $$Y_{i}=\alpha_{0}+\alpha^{\prime }X_{i}+\beta^{\prime }G_{i}+\varepsilon_{i}$$

Subsequently, the variance‐component score statistic is computed as

(6) $$Q={\left(Y-\hat{Y}\right)}^{T}\;K\left(Y-\hat{Y}\right)$$

where *K*  =  *GWG^T^*. Here, $\hat{Y}$ represents the predicted phenotype, accounting for the influence of covariates, while *G* denotes the genotype matrix for *p* variants, and *W* signifies the diagonal weights matrix associated with the minor allele frequency (MAF) for each variant within region *i*.

In this study, RECWAS leverages pre‐trained weights sourced from the CWAS model, along with marginal effects, to serve as weights in the analysis. This approach is specifically tailored to focus on prostate cancer, targeting the AR and H3K27ac for peak‐trait association analysis under a SKAT‐like protocol. In our implementation, we define a genomic region as a 25 kb span centered around each peak, in line with the SKAT framework, while deliberately omitting the consideration of covariate impacts. To assess the associations within these regions, we employed the score test

(7) $$Q=Y^{T}\;KY$$

along with a linear weighted kernel function, to construct the matrix

(8) $$K=GWG^{T}$$

which represents the inter‐individual similarity. Here, let *Y* represent the phenotype vector for *n* individuals, *G* denotes the *n* × *p* genotype matrix incorporating *p* genetic variants within the region. Statistically, we consider the following linear model

(9) $$Y_{i}=\alpha_{0}+G_{i}\beta +\alpha Z_{i}+\varepsilon_{i},\;\varepsilon_{i}∼N\left(0,\sigma_{\varepsilon }^{2}\right)$$

for continuous traits, and

(10) $$logit\;\left(Porb\left(Y_{1}=1\right)\right)=\alpha_{0}+G_{i}\;\beta +Z_{i}\alpha +\varepsilon_{i},\;\varepsilon_{i}∼N\left(0,\sigma_{\varepsilon }^{2}\right)$$

for dichotomous traits (i.e., *Y_i_* =  0/1), where ***α *** = (α_1_,….,  α_*p*_)  is the effect of covariates, and ***β *** = (β_1_,….,  β_*p*_)  is the genetic effect on trait *Y_i_*. Here, we aim to check whether this region has a genetic effect on the phenotype, i.e., test the null hypothesis ***β *** =  0.

Taking into consideration the effects ***w***_***i***_ estimated from CWAS model, we assume $\beta_{i}\;∼\;N\left(0,\;w_{i}^{2}\tau \right)$. This problem falls into the framework of variance component testing in a generalized linear model and is similar to SKAT. Now, RECWAS score statistic is defined as

(11)

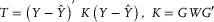

where $\hat{Y}$ is the estimated phenotype under the null model given by

(12) $$\hat{Y}=Z_{i}\;\hat{\alpha }$$

for continuous traits and

(13) $$\hat{Y}=\mathrm{logi}t^{-1}\left(Z_{i}\;\hat{\alpha }\right)$$

for dichotomous traits, $W=diag\{w_{1}^{2},w_{2}^{2},\text{…},w_{p}^{2}\}$, *G* and *Z* are assumed to be normalized to have mean zero. Since the score statistic *T* follows a mixture of chi‐squared distributions under the null hypothesis, its *p* value can be conveniently computed by approximation methods like the Davies method.^[^ ^59^ ^]^

Compared with SKAT, we solely considered the common variants for computation, while maintaining a consistent significance threshold as utilized in the CWAS analysis. Another point different from SKAT is that we actually use a kernel in the form of *GW*_1_ *W*_1_′*G*′, with $W_{1}=diag\left\{w_{1}^{2},w_{2}^{2},\text{…},w_{p}^{2}\right\}$ of the same order with the weight in SKAT, and these weights are estimated from CWAS, which is in a similar spirit to VC‐TWAS^[^ ^26^ ^]^ which takes eQTL effect size estimates as variant weight. This framework is easily extended to the semiparametric model:

(14) $$Y_{i}=\alpha_{0}+f\left(G_{i}\right)+\alpha Z_{i}+\varepsilon_{i},\;\varepsilon_{i}∼N\left(0,\sigma_{\varepsilon }^{2}\right),\;\beta_{i}\;∼\;N\left(0,\;w_{i}^{2}\tau \right)$$

for continuous traits and

(15) $$\begin{array}{ccc} logit\;\left(Porb\left(Y_{i}=1\right)\right) & = & \alpha_{0}+f\left(G_{i}\right)+Z_{i}\alpha +\varepsilon_{i},\;\varepsilon_{i}∼N\left(0,\sigma_{\varepsilon }^{2}\right),\\ & & \beta_{i}\;∼\;N\left(0,\;w_{i}^{2}\tau \right) \end{array}$$

for dichotomous traits.

Now, we are interested in testing the null hypothesis *H*_0_:  *f* (*G_i_*) =  0, under the assumption that *Ef* (*G_i_*) =  0. This can be done by assuming (*f*(*G*_1_),….*f*(*G_n_*)) follows a Gaussian distribution with mean zero and covariance τ*K*, and then tests the null hypothesis that τ  =  0 by a variance component score test. In this case, the kernel matrix *K* should have a nonlinear form taking into consideration the nonlinearity of function *f*. For example, it can be defined with elements given by

(16) $$K\;\left(G_{i},G_{j}\right)=\sum w_{j}IBS\left(G_{i},G_{j}\right)$$

### Simulation Design: Generation of Cistrome and Phenotype Data

For our study, we selected the genotype data of male individuals from the GTEx^[^ ^18^ ^]^ dataset, comprising 13 644 290 SNPs. We performed quality control by excluding SNPs with a MAF greater than 0.05 and a missing rate exceeding 0.05. Additionally, any samples exhibiting a missing rate above 0.05 were also removed. Similar preprocessing steps were applied to the 1000 Genomes Project (1KGP) data,^[^ ^60^ ^]^ which led to the retention of 441 samples and 4 500 300 genotypes from the GTEx dataset, and 1233 retained samples and 4 500 300 genotypes from the 1KGP dataset. In our simulation procedure, the GTEx dataset functioned as the reference panel, while the 1000 Genomes project dataset served as the test dataset, providing a comprehensive framework for our genetic analysis.

### Cistrome Data

In our simulation, we modeled the cistrome activity of 18 657 ARBS using real genotype data obtained from the GTEx and 1KGP datasets. For each AR peak, we defined a genomic region spanning ±25 kb centered on the peak to include relevant genetic variants. These regions were then subjected to simulations based on 5 distinct genetic architectures, encompassing both additive and non‐additive models, to comprehensively assess their genetic effects (Figure S6, Supporting Information).

In the additive genetic architecture, cistrome activity and phenotypes are simulated using a weighted sum of genetic effects. For each AR peak center, we randomly selected *n* SNPs from within a ±25 kb genomic region surrounding the peak. Each of these SNPs was assigned a weight, derived from a normal distribution, with β values following β∼N(0,1). Following this, the cistrome activity for each peak was calculated using a specific equation that integrates these weighted genetic effects, thereby providing a simulated representation of the cistrome's response to the underlying genetic variation

(17) $$C=\sum_{i=1}^{n}\beta_{i}G_{i}$$

where β_*i*_ represents the weight of genotype *G_i_*.

The non‐additive architecture in our study encompasses 4 distinct models: single, epistatic, heterogeneous, and compensatory. The single model is centered on a unique variant's contribution to the cistrome, wherein the consequent “imputed” cistrome state predominantly influences phenotype changes. This model is particularly significant in the context of CWAS, accounting for a substantial proportion of the observed changes. In contrast, the epistatic, heterogeneous, and compensatory models delve into the interactive effects of 2 randomly chosen variants on the cistrome and subsequent phenotype alterations. Specifically, the epistatic model requires both SNPs to carry mutated alleles for any change in cistrome/phenotype to manifest. The heterogeneous model, however, allows for cistrome/phenotype alterations with at least one mutated allele present in the 2 SNPs. The compensatory model presents a unique scenario where changes are triggered by a single SNP with a mutated allele, but if mutations are present in both SNPs, their effects cancel each other out, negating any change (Figure 1E). Collectively, these 3 models of nonlinear interactions address the complex regulatory dynamics between SNPs, offering a view of the intricate mechanisms underlying genetic regulation in our study.

### Phenotype Data

To facilitate a thorough comparison of CWAS and RECWAS, we performed simulation analyses focusing on 2 distinct genetic architectures: causality and pleiotropy. The causality model is based on a genotype that first influences cistrome activity and subsequently alters the phenotype. In contrast, the pleiotropy model involves a genotype that concurrently impacts both cistrome activity and phenotype, with the genotype being the causal factor for both. In each of these models, the genetic component affecting the cistrome or phenotype is simulated as a value between 0 and 1. This value is then appropriately rescaled to align with the heritability of the cistrome or phenotype, ensuring a realistic representation of the genetic effect.

### Heritability

Considering the equation

(18) Phenotype=Genotype+Enviroment

which can be expressed as

(19) $$Var\;\left(C\right)=Var\left(G\right)+Var\left(E\right)$$

when cistrome activity *C* is defined as the phenotype, we incorporated the effect of the environment or non‐genetic factors contributing to peak activity. This contribution was calculated using

(20) $$h^{2}=\frac{Var\left(G\right)}{Var\left(C\right)}$$

The non‐genetic effects were modeled by the normal distribution $C_{E}∼N\left(0,Var(E)\right)$. Consequently, the simulated peak activity in association tests and power calculations was given by *C*  =  *C_G_* + *C_E_*. In the subsequent step, the phenotype was simulated from the genotype or simulated cistrome activity following the same procedure as the previous step.

Here, we utilized cistrome and phenotype heritability to consider non‐genetic components, such as noise or environmental effects, in the cistrome and phenotype activity. This resulted in a final simulated value that can be used for association calculations and power evaluations.

### Power Calculation and Type I Error Estimation

In this study, we utilized the simulated cistrome data in conjunction with the GTEx genotype data to establish a reference panel. The panel's weights were trained using LASSO regression and marginal effects, as calculated by fastQTL,^[^ ^61^ ^]^ aiming to precisely capture the genotype's contribution to the cistrome. This approach was mirrored for the 1KGP data, where we simulated the phenotype using identical weights (*β*) as established in the GTEx dataset analysis. Consequently, for each model under consideration, the reference cistrome data were generated employing GTEx genotypes, ensuring methodological consistency across our simulations. During the testing step, we applied CWAS and RECWAS methods to explore the associations between the predicted cistrome levels and the simulated phenotypes within the 1KGP dataset.

In our simulation framework designed to mimic the impact of regulatory variants, we varied the number of variants (*n*) to 2, 3, 5, and 10, selecting only those with a MAF greater than 5%. To model the genetic influence on cistrome and phenotype, we set distinct heritability levels for the causality and pleiotropy models. For the causality model, cistrome and phenotype heritability were set at 0.05, 0.10, 0.15, 0.20, and 0.25, while for the pleiotropy model, these were adjusted to 0.01, 0.02, 0.04, 0.06, and 0.08. This strategic variation allowed for a comprehensive exploration of different genetic scenarios. Focusing on the AR model, each genetic architecture and its corresponding parameters were used to simulate 18 657 peaks. The effectiveness of each protocol—CWAS and RECWAS—in identifying these simulated peaks was rigorously tested. Success in this context was quantitatively defined as achieving a *p* value less than the threshold of 0.05/5580. This approach enabled a detailed assessment of each protocol's power in detecting significant cistrome‐phenotype associations under varying heritability conditions and genetic architectures.

To assess the type I error of CWAS and RECWAS, we simulated random phenotypes with no genetic effect. This enabled us to establish the null distribution for each method. Next, we analyzed the 18 657 peaks to determine the threshold value for each protocol, which identifies the top 5% most significant results.

### Real Data Source and QC

In addition to the simulations, we also analyzed prostate cancer real data to compare the CWAS and RECWAS protocols. Below are the sources of data and procedures for processing. First, we downloaded the OncoArray genotype data of prostate cancer^[^ ^62^ ^]^ from the dbGaP^[^ ^63^ ^]^ web portal, under the accession code phs001391.v1.p1. Subsequently, the genotype data was processed with meticulous quality control using PLINK1.9.^[^ ^64^ ^]^ Initially, we excluded variants and samples with missing rates exceeding 0.01, as well as variants with a MAF below 0.01 and those displaying significant deviations from Hardy‐Weinberg equilibrium (*p* value>1e−7). We pruned out variants exhibiting high LD using the “–indep‐pairwise 100 kb 1 0.8” command. Samples demonstrating considerable deviations from F coefficient estimates of homozygosity were filtered out using the “–het” command. Indel variants were also excluded from further analysis. The initial dataset phs001391.v1.p1.c1 contained 505 219 variants and 29 707 individuals, while the dataset phs001391.v1.p1.c3 initially comprised 505 219 variants and 31 986 individuals. After implementing quality control measures, phs001391.v1.p1.c1 had 305 867 variants and 28 554 individuals, while phs001391.v1.p1.c3 had 326 352 variants and 30 450 individuals.

### Validation Analysis of Associations

In our study, we conducted a comprehensive analysis to rigorously evaluate the reliability and biological significance of the results obtained from the CWAS and RECWAS protocols. This involved a series of meticulous analyses, each incorporating multiple forms of biological evidence.

First, we established a robust set of “prostate cancer gold peak datasets”, meticulously defined to encompass significant peaks closely associated with prostate cancer. This compilation was derived from CWAS association tests, utilizing GWAS summary statistics from an extensive cohort of 140 306 males.^[^ ^62^ ^]^ Notably, this dataset comprised 74 ARBS and 199 H3k27ac peaks related to prostate cancer risk, which was computed by CWAS and extracted from Tables (S6 and S7) in the study of Baca et al.,^[^ ^37^ ^]^ The aim was to quantitatively assess the overlap of significant peaks identified by both CWAS and RECWAS with these gold standard datasets, providing a vital benchmark for comparison.

Further, we leveraged the GWAS Catalog,^[^ ^65^ ^]^ a curated repository of SNP‐trait associations from GWAS, to extract genome‐wide significant SNPs (*p* value < 1e‐05) linked to “prostate carcinoma”. This search yielded a comprehensive list of SNPs, which were assigned hg19 coordinates. A genomic region of ±1Mb surrounding each SNP was designated as a GWAS risk region, culminating in a total of 261 distinct prostate cancer risk regions for in‐depth analysis.

Additionally, the DisGeNET database,^[^ ^38^ ^]^ renowned for its compilation of genes associated with diseases from a variety of public databases and literature mining, was utilized. A search with the keyword “prostate carcinoma” yielded a substantial list of 4222 prostate cancer‐associated genes. These genes were mapped to hg19 coordinates, and a proximity window of 100 kb on either side of these genes was defined as the prostate cancer susceptibility‐related region. This enabled a comprehensive examination of the overlap of significant peaks identified by CWAS and RECWAS with both GWAS risk regions and prostate cancer susceptibility‐related regions. Simultaneously, the shortest distance of each significant peak to the nearest prostate cancer‐associated gene was meticulously calculated.

In addition to these analyses, we also investigated genes with EDS, as listed in Table S1 in the study of Wang et al.,^[^ ^39^ ^]^ encompassing a total of 20 065 genes. EDS scores serve as indicators of the extent of regulatory DNA redundancy for each gene, based on the number of predicted enhancers and the redundancy of transcription factor motifs within them. For each of these genes, a genomic region of ±100 kb was set, and an overlapping analysis was conducted with the significant peaks identified by CWAS and RECWAS. This rigorous approach provided a deeper insight into the regulatory potential of these significant peaks, offering a comprehensive view of their roles in the intricate landscape of genetic regulation in prostate cancer.

## Conflict of Interest

The authors declare no conflict of interest.

## Author Contributions

M.S., M.T., K.C., and H.J. contributed equally to this work. N.G. and C.C. administered and designed the study and acquired funding. M.S. performed data analysis and drafted the manuscript. M.T., K.C., and H.J. discussed the results and wrote the original draft. S.Z., Z.L., Y.S., F.C., and B.S. revised the manuscript. All authors reviewed, revised, and approved the manuscript.

## Supporting information

Supporting Information

## Data Availability

The data that support the findings of this study are available in the supplementary material of this article. PLINK, https://www.cog‐genomics.org/plink/. Prostate Cancer data is publicly available and can be obtained via authorized access from the dbGAP data portal (dbGaP Study Accession: phs001391.v1.p1), https://www.ncbi.nlm.nih.gov/projects/gap/cgi‐bin/study.cgi?study_id=phs001391.v1.p1. Myocardial infarction, venous thrombosis, melanoma, oral and pharynx cancer data is publicly available and can be obtained via authorized access from the dbGAP data portal (dbGaP Study Accession: phs000294.v1.p1.c1, phs000289.v2.p1.c1, phs000187.v1.p1.c1, phs001202.v2.p1.c1), https://www.ncbi.nlm.nih.gov/projects/gap/cgi‐bin/study.cgi?study_id=phs000294.v1.p1.c1, https://www.ncbi.nlm.nih.gov/projects/gap/cgi‐bin/study.cgi?study_id=phs000289.v2.p1.c1, https://www.ncbi.nlm.nih.gov/projects/gap/cgi‐bin/study.cgi?study_id=phs000187.v1.p1.c1, https://www.ncbi.nlm.nih.gov/projects/gap/cgi‐bin/study.cgi?study_id=phs001202.v2.p1.c1. The GTEx individual‐level genotypes were obtained from the dbGaP (dbGaP Study Accession: phs000424.v9.p2), https://www.ncbi.nlm.nih.gov/projects/gap/cgi‐bin/study.cgi?study_id=phs000424.v9.p2. 1000 Genomes Project, https://www.internationalgenome.org/. The RECWAS software is publicly available on GitHub (https://github.com/PrecisionWAS/RECWAS), accepting individual genotype data of tfam, tped format as input, and all necessary files and packages were integrated into a jar executable file. Additionally, users should install the R Statistical Software v4.3.1 (https://www.R‐project.org/) and SKAT[58] package (https://CRAN.R‐project.org/package=SKAT).
