# Supplementary material for: Leveraging Random Effects in Cistrome‐Wide Association Studies for Decoding the Genetic Determinants of Prostate Cancer
Source: Adv Sci (Weinh). 2024 Aug 5;11(36):2400815. doi: 10.1002/advs.202400815 (PMC11423091; doi:10.1002/advs.202400815)
Supplement: Supplementary file 1 — Supporting Information [file ADVS-11-2400815-s001.docx]

# Supporting Information

**Leveraging Random Effects in Cistrome-Wide Association Studies for Decoding the Genetic Determinants of Prostate Cancer**

Mengting Shao^1^, Min Tian^1^, Kaiyang Chen^1^, Hangjin Jiang^2^, Shuting Zhang^1^, Zhenghui Li^1^, Yan Shen^1^, Feng Chen^1^, Baixin Shen^3^, Chen Cao^1,3,*^, Ning Gu^1,4,*^

^1^ Key Laboratory for Bio-Electromagnetic Environment and Advanced Medical Theranostics, School of Biomedical Engineering and Informatics, Nanjing Medical University, Nanjing 211166, P.R. China

^2^ Center for Data Science, Zhejiang University, Hangzhou 310058, P.R. China

^3^ Department of Urology, The Second Affiliated Hospital of Nanjing Medical University, Nanjing 210011, P.R. China

^4^ Nanjing Key Laboratory for Cardiovascular Information and Health Engineering Medicine, Institute of Clinical Medicine, Nanjing Drum Tower Hospital, Medical School, Nanjing University, Nanjing 210093, P.R. China

^*^ Corresponding author

Correspondence should be addressed to C.C. (caochen@njmu.edu.cn) and N.G. (guning@nju.edu.cn).

Table S1. Detail information about significant AR peaks identified by CWAS or RECWAS.

| **Peak** | **CWAS P-value** | **RECWAS P-value** | **Status** | **In putative** | **In GWAS catalog** | **Closest gene** | **Closest gene in DisGeNET** | **Mapped DisGeNET gene** | **Distance** |
| --- | --- | --- | --- | --- | --- | --- | --- | --- | --- |
| chr1:184435000-184435400 | 9.18E-03 | 1.21E-07 | CWAS- RECWAS+ | - | - | C1orf21 | - | RNF2 | 579096 |
| chr1:205794050-205794700 | 5.56E-03 | 1.56E-07 | CWAS- RECWAS+ | - | - | PM20D1 | - | SLC45A3 | 144463 |
| chr11:21586300-21586950 | 2.45E-06 | 1.48E-05 | CWAS+ RECWAS- | - | - | NELL1 | - | HTATIP2 | 1180971 |
| chr11:2223450-2224100 | 2.21E-14 | 3.41E-14 | CWAS+ RECWAS+ | - | + | MIR4686 | - | TH | 30343 |
| chr11:2243550-2244250 | 1.27E-03 | 7.42E-08 | CWAS- RECWAS+ | - | + | ASCL2 | - | TH | 50443 |
| chr11:68882950-68883500 | 2.53E-12 | 9.39E-18 | CWAS+ RECWAS+ | - | + | RP11-554A11.7 | - | TPCN2 | 24878 |
| chr11:68890000-68890700 | 1.12E-06 | 1.22E-09 | CWAS+ RECWAS+ | - | + | RP11-554A11.7 | - | TPCN2 | 31928 |
| chr11:7606050-7606650 | 1.05E-05 | 4.87E-06 | CWAS- RECWAS+ | - | + | PPFIBP2 | + | PPFIBP2 | 71521 |
| chr12:3343000-3343550 | 9.00E-07 | 3.18E-04 | CWAS+ RECWAS- | - | - | TSPAN9 | - | FOXM1 | 356794 |
| chr13:91993700-91994350 | 4.55E-06 | 5.05E-06 | CWAS+ RECWAS+ | - | - | MIR17HG | + | MIR17HG | 5724 |
| chr14:52880350-52881250 | 5.84E-07 | 1.08E-05 | CWAS+ RECWAS- | - | + | TXNDC16 | - | PTGER2 | 85026 |
| chr16:79842850-79843900 | 5.77E-07 | 1.18E-04 | CWAS+ RECWAS- | - | - | RP11-345M22.3 | - | WWOX | 596286 |
| chr17:17875700-17876350 | 1.40E-08 | 1.69E-08 | CWAS+ RECWAS+ | - | - | TOM1L2 | - | SREBF1 | 135375 |
| chr17:17942400-17942800 | 6.75E-09 | 3.18E-08 | CWAS+ RECWAS+ | - | - | ATPAF2 | - | ALKBH5 | 143592 |
| chr17:36003300-36003750 | 3.09E-07 | 7.99E-09 | CWAS+ RECWAS+ | - | + | RP11-697E22.2 | - | DDX52 | 193 |
| chr17:36129450-36129950 | 1.17E-28 | 5.71E-31 | CWAS+ RECWAS+ | - | + | RP11-115K3.1 | - | HNF1B | 24213 |
| chr19:51339200-51339600 | 5.14E-02 | 1.96E-07 | CWAS- RECWAS+ | - | + | KLK15 | + | KLK15 | 869 |
| chr19:51345150-51346200 | 5.75E-06 | 2.74E-18 | CWAS+ RECWAS+ | - | + | AC011523.2 | - | KLK15 | 4681 |
| chr19:51364150-51365300 | 5.75E-06 | 3.97E-08 | CWAS+ RECWAS+ | - | + | KLK3 | + | KLK3 | 130 |
| chr19:51372350-51373300 | 6.65E-32 | 1.57E-34 | CWAS+ RECWAS+ | - | + | KLK2 | + | KLK2 | 7526 |
| chr2:63084250-63084850 | 3.78E-07 | 3.86E-07 | CWAS+ RECWAS+ | - | + | EHBP1 | + | EHBP1 | 183264 |
| chr2:80117500-80118150 | 1.70E-06 | 3.93E-04 | CWAS+ RECWAS- | - | - | CTNNA2 | - | REG3A | 730621 |
| chr20:52402400-52403950 | 5.69E-06 | 1.13E-06 | CWAS+ RECWAS+ | - | + | AC006076.1 | - | BCAS1 | 149366 |
| chr20:52444350-52445650 | 4.81E-05 | 6.59E-06 | CWAS- RECWAS+ | + | + | AC006076.1 | - | BCAS1 | 107666 |
| chr20:52468500-52469200 | 6.48E-07 | 6.75E-07 | CWAS+ RECWAS+ | - | + | SUMO1P1 | - | BCAS1 | 84116 |
| chr20:54243000-54243450 | 2.10E-06 | 3.43E-03 | CWAS+ RECWAS- | - | - | RP5-1010E17.1 | - | AURKA | 700995 |
| chr3:128065300-128066100 | 2.02E-12 | 3.59E-12 | CWAS+ RECWAS+ | + | + | EEFSEC | + | EEFSEC | 61385 |
| chr3:23156500-23157200 | 4.02E-04 | 6.02E-06 | CWAS- RECWAS+ | - | - | RPL24P7 | - | THRB | 1001451 |
| chr3:23171200-23171900 | 1.24E-02 | 3.74E-06 | CWAS- RECWAS+ | - | - | RPL24P7 | - | THRB | 986751 |
| chr3:88107900-88108350 | 7.13E-06 | 9.41E-06 | CWAS+ RECWAS- | - | + | CGGBP1 | - | EPHA3 | 1048324 |
| chr4:106065150-106065500 | 3.13E-11 | 3.84E-11 | CWAS+ RECWAS+ | - | + | TET2 | + | TET2 | 1532 |
| chr4:152094650-152095450 | 1.79E-06 | 4.74E-06 | CWAS+ RECWAS+ | - | - | SH3D19 | - | FBXW7 | 1146960 |
| chr4:77129450-77130050 | 5.72E-06 | 1.02E-05 | CWAS+ RECWAS- | - | - | SCARB2 | + | SCARB2 | 4996 |
| chr6:117122900-117123750 | 1.55E-06 | 1.79E-06 | CWAS+ RECWAS+ | + | + | GPRC6A | + | GPRC6A | 9652 |
| chr6:160561950-160562800 | 1.73E-02 | 6.20E-06 | CWAS- RECWAS+ | - | + | SLC22A1 | + | SLC22A1 | 16950 |
| chr6:160659400-160660100 | 4.60E-09 | 1.53E-12 | CWAS+ RECWAS+ | + | + | SLC22A2 | - | SLC22A1 | 79650 |
| chr6:160785700-160786300 | 7.33E-06 | 7.30E-06 | CWAS+ RECWAS+ | - | + | SLC22A3 | + | SLC22A3 | 16400 |
| chr6:160833300-160833900 | 7.49E-07 | 8.80E-07 | CWAS+ RECWAS+ | - | + | SLC22A3 | + | SLC22A3 | 42114 |
| chr6:31243050-31243450 | 1.23E-01 | 3.49E-06 | CWAS- RECWAS+ | - | + | USP8P1 | - | HLA-C | 3143 |
| chr7:151112500-151112800 | 2.61E-05 | 5.05E-06 | CWAS- RECWAS+ | - | - | WDR86-AS1 | - | RHEB | 50298 |
| chr7:97855250-97856000 | 4.59E-07 | 5.59E-08 | CWAS+ RECWAS+ | - | + | TECPR1 | - | LMTK2 | 16305 |
| chr8:127877350-127877700 | 3.89E-04 | 6.26E-07 | CWAS- RECWAS+ | - | + | PCAT1 | + | PCAT1 | 18050 |
| chr8:127888700-127889300 | 4.16E-13 | 4.52E-18 | CWAS+ RECWAS+ | - | + | PCAT1 | + | PCAT1 | 29400 |
| chr8:127938850-127939600 | 1.20E-05 | 2.89E-06 | CWAS- RECWAS+ | - | + | PCAT1 | + | PCAT1 | 79550 |
| chr8:128034550-128035150 | 1.27E-05 | 1.18E-19 | CWAS- RECWAS+ | - | + | PCAT1 | + | PCAT1 | 1291 |
| chr8:128064750-128065250 | 4.80E-06 | 2.21E-09 | CWAS+ RECWAS+ | - | + | PCAT2 | + | PCAT2 | 19689 |
| chr8:128283100-128283650 | 7.40E-02 | 6.95E-06 | CWAS- RECWAS+ | - | + | CASC8 | + | CASC8 | 18412 |
| chr8:128333350-128334000 | 4.63E-03 | 1.04E-11 | CWAS- RECWAS+ | - | + | CASC8 | + | CASC8 | 31288 |
| chr8:128342400-128343150 | 3.93E-19 | 3.19E-15 | CWAS+ RECWAS+ | + | + | CASC8 | + | CASC8 | 40338 |
| chr8:128410300-128410950 | 1.54E-10 | 4.10E-23 | CWAS+ RECWAS+ | - | + | CASC8 | + | CASC8 | 83434 |
| chr8:128413000-128413900 | 5.65E-52 | 1.08E-44 | CWAS+ RECWAS+ | + | + | CASC8 | + | CASC8 | 80484 |
| chr8:128420500-128421200 | 1.50E-02 | 4.07E-32 | CWAS- RECWAS+ | + | + | CASC8 | + | CASC8 | 73184 |
| chr8:128440700-128441300 | 1.50E-02 | 1.50E-11 | CWAS- RECWAS+ | + | + | CASC8 | + | CASC8 | 53084 |
| chr8:128519450-128520550 | 7.14E-46 | 7.45E-35 | CWAS+ RECWAS+ | - | + | CASC8 | + | CASC8 | 25066 |
| chr8:128521850-128522850 | 1.54E-67 | 1.13E-57 | CWAS+ RECWAS+ | - | + | CASC8 | + | CASC8 | 27466 |
| chr8:128531150-128532050 | 1.54E-67 | 7.47E-60 | CWAS+ RECWAS+ | + | + | CASC8 | + | CASC8 | 36766 |
| chr9:35116100-35116500 | 1.44E-07 | 2.74E-06 | CWAS+ RECWAS+ | - | - | FAM214B | - | VCP | 42854 |

Table S2. Detail information about significant H3k27ac peaks identified by CWAS or RECWAS.

| **Peak** | **CWAS P-value** | **RECWAS P-value** | **Status** | **In putative** | **In GWAS catalog** | **Closest gene** | **Closest gene in DisGeNET** | **Mapped DisGeNET gene** | **Distance** |
| --- | --- | --- | --- | --- | --- | --- | --- | --- | --- |
| chr1:154134450-154134800 | 3.67E-06 | 2.09E-06 | CWAS- RECWAS+ | - | + | TPM3 | - | UBAP2L | 57855 |
| chr1:154984200-154984600 | 2.02E-06 | 2.01E-06 | CWAS+ RECWAS+ | - | + | ZBTB7B | - | FLAD1 | 18613 |
| chr1:154989100-154990700 | 2.02E-06 | 2.01E-06 | CWAS+ RECWAS+ | - | + | ZBTB7B | - | FLAD1 | 23513 |
| chr1:154995100-154996100 | 2.02E-06 | 2.01E-06 | CWAS+ RECWAS+ | - | + | DCST2 | - | ADAM15 | 26942 |
| chr1:184333550-184334050 | 1.64E-06 | 1.43E-06 | CWAS+ RECWAS+ | - | - | RP11-382D12.2 | - | RNF2 | 680446 |
| chr1:184412100-184413200 | 4.50E-08 | 6.27E-08 | CWAS+ RECWAS+ | - | - | C1orf21 | - | RNF2 | 601296 |
| chr1:184451600-184454050 | 9.18E-03 | 1.30E-07 | CWAS- RECWAS+ | - | - | C1orf21 | - | RNF2 | 560446 |
| chr1:205752550-205753750 | 4.33E-08 | 8.98E-08 | CWAS+ RECWAS+ | - | - | SLC41A1 | - | SLC45A3 | 102963 |
| chr1:205781300-205783250 | 2.88E-06 | 1.44E-07 | CWAS+ RECWAS+ | - | - | SLC41A1 | - | SLC45A3 | 131713 |
| chr11:113980650-113981750 | 3.49E-07 | 2.76E-03 | CWAS+ RECWAS- | - | + | ZBTB16 | + | ZBTB16 | 50335 |
| chr11:2222900-2225000 | 1.27E-03 | 1.56E-10 | CWAS- RECWAS+ | + | + | MIR4686 | - | TH | 29793 |
| chr11:2229700-2231000 | 2.21E-14 | 7.10E-14 | CWAS+ RECWAS+ | + | + | MIR4686 | - | TH | 36593 |
| chr11:2232000-2232450 | 2.21E-14 | 9.22E-16 | CWAS+ RECWAS+ | + | + | MIR4686 | - | TH | 38893 |
| chr11:2232700-2233050 | 2.21E-14 | 6.21E-16 | CWAS+ RECWAS+ | + | + | MIR4686 | - | TH | 39593 |
| chr11:2243250-2243900 | 2.21E-14 | 2.04E-15 | CWAS+ RECWAS+ | + | + | ASCL2 | - | TH | 50143 |
| chr11:68853350-68853800 | 6.04E-09 | 3.92E-10 | CWAS+ RECWAS+ | + | + | TPCN2 | + | TPCN2 | 4272 |
| chr11:68872000-68875700 | 6.04E-09 | 3.41E-14 | CWAS+ RECWAS+ | - | + | RP11-554A11.7 | - | TPCN2 | 13928 |
| chr11:68881500-68883850 | 2.53E-12 | 1.08E-16 | CWAS+ RECWAS+ | + | + | RP11-554A11.7 | - | TPCN2 | 23428 |
| chr11:68924250-68925450 | 7.11E-05 | 2.23E-08 | CWAS- RECWAS+ | - | + | RP11-554A11.8 | - | TPCN2 | 66178 |
| chr11:68933650-68934650 | 1.12E-06 | 1.10E-10 | CWAS+ RECWAS+ | + | + | RP11-554A11.8 | - | TPCN2 | 75578 |
| chr11:69019000-69020750 | 1.61E-03 | 5.77E-29 | CWAS- RECWAS+ | + | + | MYEOV | - | TPCN2 | 160928 |
| chr12:58334450-58336250 | 1.15E-07 | 3.36E-04 | CWAS+ RECWAS- | - | - | XRCC6BP1 | - | CYP27B1 | 171681 |
| chr14:54420250-54421150 | 1.61E-06 | 1.61E-03 | CWAS+ RECWAS- | - | - | BMP4 | + | BMP4 | 3796 |
| chr14:69139450-69139800 | 7.46E-07 | 7.54E-07 | CWAS+ RECWAS+ | - | + | RAD51B | + | RAD51B | 57135 |
| chr14:69148850-69150550 | 7.46E-07 | 4.54E-06 | CWAS+ RECWAS- | - | + | RAD51B | + | RAD51B | 46385 |
| chr15:28365350-28366000 | 1.58E-06 | 3.90E-05 | CWAS+ RECWAS- | - | - | HERC2 | - | UBE3A | 2681222 |
| chr15:33418900-33419400 | 5.88E-03 | 2.68E-06 | CWAS- RECWAS+ | - | - | FMN1 | - | GREM1 | 392030 |
| chr16:57635150-57636150 | 1.33E-06 | 1.54E-06 | CWAS+ RECWAS+ | - | + | HMGB3P32 | - | CX3CL1 | 216190 |
| chr17:17853250-17854800 | 1.40E-08 | 1.69E-08 | CWAS+ RECWAS+ | - | - | TOM1L2 | - | SREBF1 | 112925 |
| chr17:17873600-17875900 | 1.40E-08 | 1.69E-08 | CWAS+ RECWAS+ | - | - | TOM1L2 | - | SREBF1 | 133275 |
| chr17:17941850-17943900 | 6.75E-09 | 6.57E-09 | CWAS+ RECWAS+ | - | - | ATPAF2 | - | ALKBH5 | 142492 |
| chr17:35873700-35874400 | 2.01E-06 | 3.27E-05 | CWAS+ RECWAS- | - | + | DUSP14 | - | DDX52 | 95387 |
| chr17:36002600-36004150 | 2.59E-06 | 5.09E-06 | CWAS+ RECWAS- | - | + | RP11-697E22.2 | - | DDX52 | 657 |
| chr17:36069800-36071250 | 9.28E-01 | 2.74E-07 | CWAS- RECWAS+ | - | + | HNF1B | + | HNF1B | 23365 |
| chr17:36101700-36105750 | 6.47E-52 | 2.38E-57 | CWAS+ RECWAS+ | - | + | HNF1B | + | HNF1B | 513 |
| chr17:36111500-36112050 | 2.76E-05 | 5.52E-54 | CWAS- RECWAS+ | - | + | RP11-115K3.1 | - | HNF1B | 6263 |
| chr17:7451650-7453100 | 1.69E-09 | 1.44E-09 | CWAS+ RECWAS+ | - | + | TNFSF12 | + | TNFSF12 | 558 |
| chr18:55373100-55374300 | 2.91E-06 | 2.66E-06 | CWAS- RECWAS+ | - | - | RP11-35G9.3 | - | FECH | 119096 |
| chr19:51338150-51365400 | 4.20E-09 | 6.84E-13 | CWAS+ RECWAS+ | - | + | KLK2 | + | KLK2 | 576 |
| chr19:51370450-51384750 | 3.54E-02 | 4.30E-07 | CWAS- RECWAS+ | - | + | KLKP1 | + | KLKP1 | 602 |
| chr2:201238350-201239050 | 6.05E-01 | 1.56E-09 | CWAS- RECWAS+ | - | - | SPATS2L | - | AOX1 | 211541 |
| chr20:62370150-62371050 | 1.93E-05 | 1.97E-07 | CWAS- RECWAS+ | - | + | RP4-583P15.14 | - | SLC2A4RG | 164 |
| chr20:62371100-62373250 | 3.10E-10 | 8.90E-08 | CWAS+ RECWAS+ | - | + | SLC2A4RG | + | SLC2A4RG | 114 |
| chr20:62374350-62374800 | 4.98E-14 | 1.20E-14 | CWAS+ RECWAS+ | - | + | SLC2A4RG | + | SLC2A4RG | 58 |
| chr21:40284550-40286050 | 3.65E-05 | 1.13E-06 | CWAS- RECWAS+ | - | - | AF064858.6 | - | ETS2 | 87671 |
| chr21:40293300-40293950 | 5.34E-08 | 1.34E-06 | CWAS+ RECWAS+ | - | - | AF064858.6 | - | ETS2 | 96421 |
| chr21:40297350-40298600 | 1.04E-03 | 2.01E-06 | CWAS- RECWAS+ | - | - | AF064858.6 | - | ETS2 | 100471 |
| chr21:42921100-42921550 | 1.53E-06 | 3.87E-06 | CWAS+ RECWAS- | - | + | AP001610.9 | - | TMPRSS2 | 18057 |
| chr22:43499050-43501050 | 3.93E-21 | 1.09E-20 | CWAS+ RECWAS+ | + | + | BIK | + | BIK | 5704 |
| chr3:128038800-128039350 | 2.02E-12 | 3.59E-12 | CWAS+ RECWAS+ | + | + | EEFSEC | + | EEFSEC | 88135 |
| chr3:128052400-128059200 | 2.02E-12 | 3.59E-12 | CWAS+ RECWAS+ | - | + | EEFSEC | + | EEFSEC | 68285 |
| chr3:128061900-128063250 | 2.02E-12 | 3.59E-12 | CWAS+ RECWAS+ | - | + | EEFSEC | + | EEFSEC | 64235 |
| chr3:169762100-169765450 | 2.84E-06 | 2.22E-06 | CWAS+ RECWAS+ | - | + | GPR160 | + | GPR160 | 6383 |
| chr3:169766900-169768600 | 2.84E-06 | 2.52E-06 | CWAS+ RECWAS+ | - | + | GPR160 | + | GPR160 | 11183 |
| chr4:106065650-106066100 | 3.13E-11 | 2.50E-11 | CWAS+ RECWAS+ | + | + | TET2 | + | TET2 | 932 |
| chr5:1314850-1317550 | 3.60E-08 | 6.34E-08 | CWAS+ RECWAS+ | - | + | CLPTM1L | + | CLPTM1L | 309 |
| chr6:117086000-117086900 | 1.55E-06 | 1.79E-06 | CWAS+ RECWAS+ | - | + | FAM162B | - | GPRC6A | 26348 |
| chr6:160658800-160660500 | 7.44E-02 | 8.86E-08 | CWAS- RECWAS+ | - | + | SLC22A2 | - | SLC22A1 | 79050 |
| chr6:160670300-160671500 | 2.01E-03 | 5.74E-11 | CWAS- RECWAS+ | - | + | SLC22A2 | - | SLC22A1 | 90550 |
| chr6:160815100-160816350 | 1.31E-08 | 1.79E-08 | CWAS+ RECWAS+ | - | + | SLC22A3 | + | SLC22A3 | 45800 |
| chr6:160839000-160840300 | 1.31E-08 | 1.09E-08 | CWAS+ RECWAS+ | - | + | SLC22A3 | + | SLC22A3 | 35714 |
| chr6:31238650-31240900 | 2.13E-06 | 1.91E-06 | CWAS- RECWAS+ | + | + | HLA-C | + | HLA-C | 993 |
| chr7:152596400-152597550 | 4.05E-07 | 1.53E-06 | CWAS+ RECWAS+ | - | - | ACTR3B | - | XRCC2 | 223150 |
| chr7:97840250-97844100 | 4.46E-07 | 4.52E-08 | CWAS+ RECWAS+ | + | + | TECPR1 | - | LMTK2 | 1305 |
| chr7:97846800-97847950 | 4.46E-07 | 5.15E-08 | CWAS+ RECWAS+ | + | + | TECPR1 | - | LMTK2 | 7855 |
| chr7:97852250-97854200 | 4.46E-07 | 2.20E-08 | CWAS+ RECWAS+ | + | + | TECPR1 | - | LMTK2 | 13305 |
| chr7:97854900-97856350 | 4.59E-07 | 6.13E-08 | CWAS+ RECWAS+ | - | + | TECPR1 | - | LMTK2 | 15955 |
| chr7:97887700-97888100 | 1.12E-09 | 2.57E-07 | CWAS+ RECWAS+ | - | + | BRI3 | - | LMTK2 | 48755 |
| chr8:127980950-127981600 | 7.84E-01 | 1.16E-07 | CWAS- RECWAS+ | - | + | PCAT1 | + | PCAT1 | 51659 |
| chr8:128022650-128024750 | 2.19E-02 | 5.12E-15 | CWAS- RECWAS+ | + | + | PCAT1 | + | PCAT1 | 8509 |
| chr8:128025300-128027050 | 4.06E-02 | 1.63E-17 | CWAS- RECWAS+ | - | + | PCAT1 | + | PCAT1 | 6209 |
| chr8:128027150-128034900 | 2.97E-01 | 5.27E-15 | CWAS- RECWAS+ | - | + | PCAT1 | + | PCAT1 | 1641 |
| chr8:128034950-128037250 | 4.06E-02 | 3.27E-10 | CWAS- RECWAS+ | - | + | PCAT1 | + | PCAT1 | 1691 |
| chr8:128050450-128051050 | 2.52E-02 | 8.62E-08 | CWAS- RECWAS+ | - | + | PCAT1 | + | PCAT1 | 17191 |
| chr8:128053650-128054850 | 9.91E-12 | 4.01E-15 | CWAS+ RECWAS+ | - | + | PCAT1 | + | PCAT1 | 20391 |
| chr8:128055800-128056400 | 2.52E-02 | 1.36E-07 | CWAS- RECWAS+ | - | + | PCAT1 | + | PCAT1 | 22541 |
| chr8:128064350-128066700 | 2.97E-01 | 1.76E-08 | CWAS- RECWAS+ | - | + | PCAT2 | + | PCAT2 | 18239 |
| chr8:128073500-128076150 | 2.52E-02 | 1.78E-11 | CWAS- RECWAS+ | - | + | PCAT2 | + | PCAT2 | 8789 |
| chr8:128100750-128106650 | 1.12E-05 | 4.48E-18 | CWAS- RECWAS+ | - | + | PRNCR1 | + | PRNCR1 | 995 |
| chr8:128109200-128115550 | 4.21E-03 | 1.43E-07 | CWAS- RECWAS+ | - | + | PRNCR1 | + | PRNCR1 | 9445 |
| chr8:128289000-128289900 | 2.01E-10 | 3.12E-10 | CWAS+ RECWAS+ | - | + | CASC8 | + | CASC8 | 12162 |
| chr8:128333100-128333850 | 3.93E-19 | 5.76E-15 | CWAS+ RECWAS+ | - | + | CASC8 | + | CASC8 | 31038 |
| chr8:128341450-128343650 | 3.51E-01 | 9.72E-07 | CWAS- RECWAS+ | - | + | CASC8 | + | CASC8 | 39388 |
| chr8:128412550-128415150 | 9.56E-07 | 2.15E-27 | CWAS+ RECWAS+ | - | + | CASC8 | + | CASC8 | 79234 |
| chr8:128490400-128491800 | 1.42E-32 | 1.42E-37 | CWAS+ RECWAS+ | - | + | CASC8 | + | CASC8 | 2584 |
| chr8:128494150-128494650 | 3.02E-04 | 7.62E-34 | CWAS- RECWAS+ | - | + | CASC8 | + | CASC8 | 234 |
| chr8:128504700-128507650 | 7.82E-08 | 1.61E-10 | CWAS+ RECWAS+ | + | + | CASC8 | + | CASC8 | 10316 |
| chr8:128519000-128522100 | 7.14E-46 | 6.22E-44 | CWAS+ RECWAS+ | - | + | CASC8 | + | CASC8 | 24616 |
| chr8:128530900-128531550 | 1.53E-67 | 4.48E-61 | CWAS+ RECWAS+ | - | + | CASC8 | + | CASC8 | 36516 |
| chr9:110151550-110152950 | 3.98E-06 | 1.10E-06 | CWAS- RECWAS+ | + | + | RP11-363D24.1 | - | RAD23B | 57075 |
| chr9:18554400-18555350 | 1.62E-05 | 7.93E-07 | CWAS- RECWAS+ | + | + | ADAMTSL1 | - | RPS6 | 820363 |
| chr9:35102150-35104050 | 1.44E-07 | 2.74E-06 | CWAS+ RECWAS+ | - | - | FAM214B | - | VCP | 28904 |
| chr9:35111750-35112250 | 1.44E-07 | 2.74E-06 | CWAS+ RECWAS+ | - | - | FAM214B | - | VCP | 38504 |
| chr9:35114200-35117650 | 1.44E-07 | 2.74E-06 | CWAS+ RECWAS+ | - | - | FAM214B | - | VCP | 40954 |
| chr9:35127100-35127600 | 1.44E-07 | 2.74E-06 | CWAS+ RECWAS+ | - | - | FAM214B | - | VCP | 53854 |

Table S3. Information about non-prostate cancer datasets.

| dbGaP Study Accession | Number of Sample | Number of SNPs | Trait | Number of significant peaks identified by CWAS | Number of significant peaks identified by RECWAS |
| --- | --- | --- | --- | --- | --- |
| phs000294.v1.p1.c1 | 5862 | 359404 | Myocardial infarction | 1 | 1 |
| phs000289.v2.p1.c1 | 2566 | 383516 | Venous Thrombosis | 1 | 2 |
| phs000187.v1.p1.c1 | 2991 | 497986 | Melanoma | 1 | 3 |
| phs000351.v1.p1.c1 | 3255 | 375749 | Renal Cell Carcinoma | 721 | 1053 |
| phs001202.v2.p1.c1 | 8369 | 341153 | Oral and Pharynx Cancer | 658 | 665 |


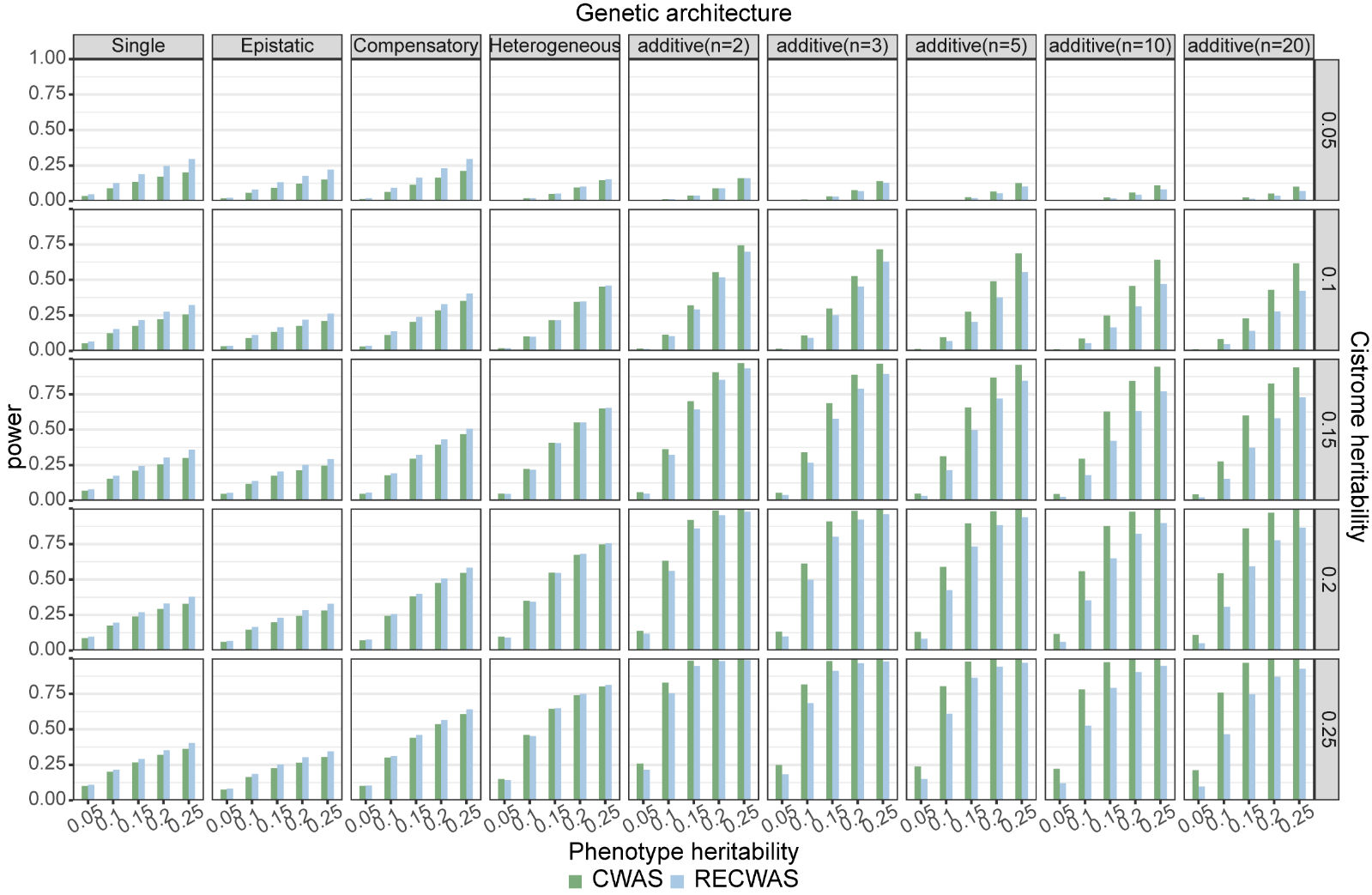


Figure S1. Statistical power (left-y-axis) of CWAS and RECWAS method compared on causality model simulated at varying levels of cistrome heritability (right-y-axis), phenotype heritability (x-axis), and different genetic architectures including single, epistatic, compensatory, heterogeneous, and additive.


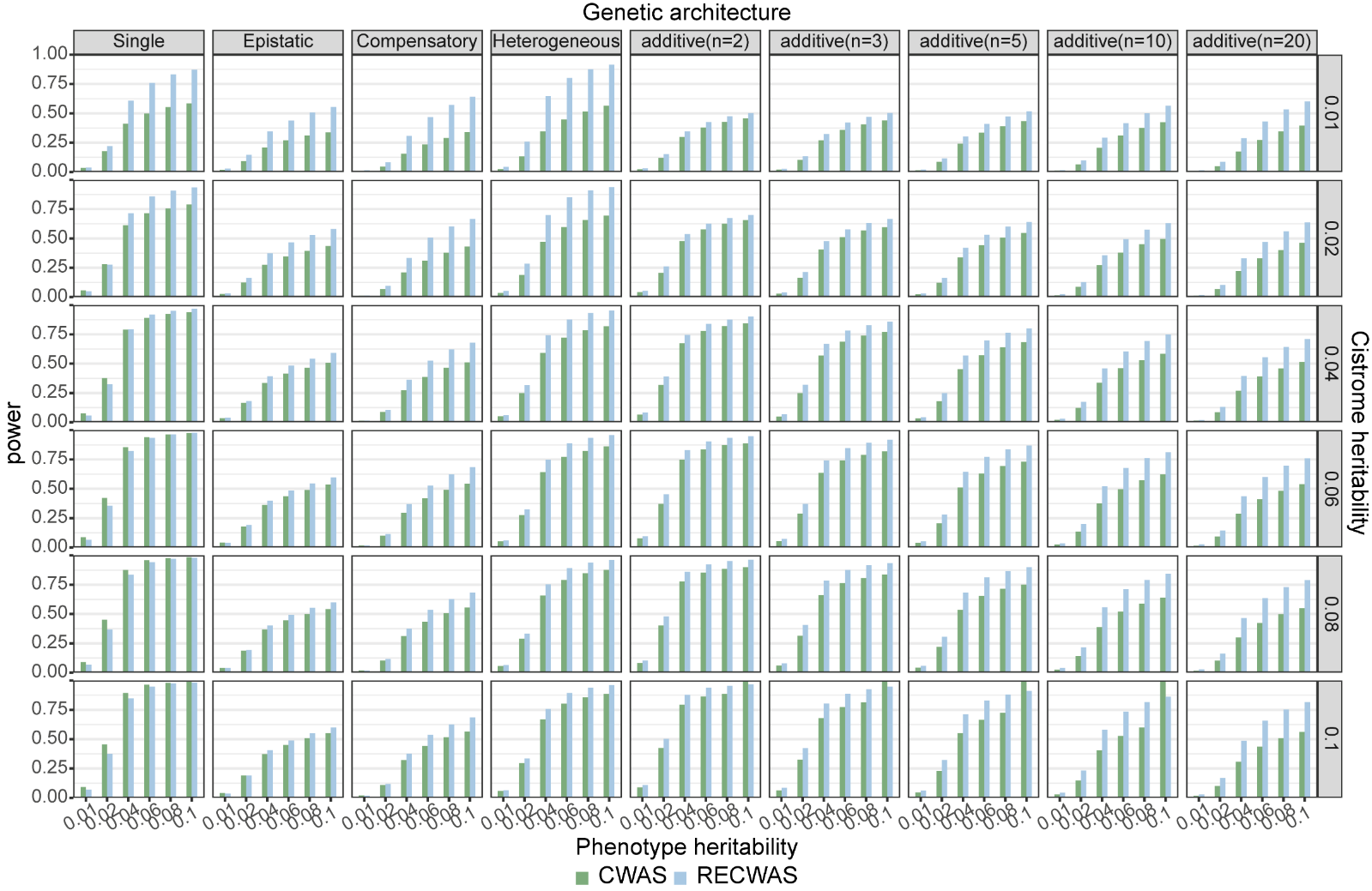


Figure S2. Statistical power (left-y-axis) of CWAS and RECWAS method compared on pleiotropy model simulated at varying levels of cistrome heritability (right-y-axis), phenotype heritability (x-axis), and different genetic architectures including single, epistatic, compensatory, heterogeneous, and additive.


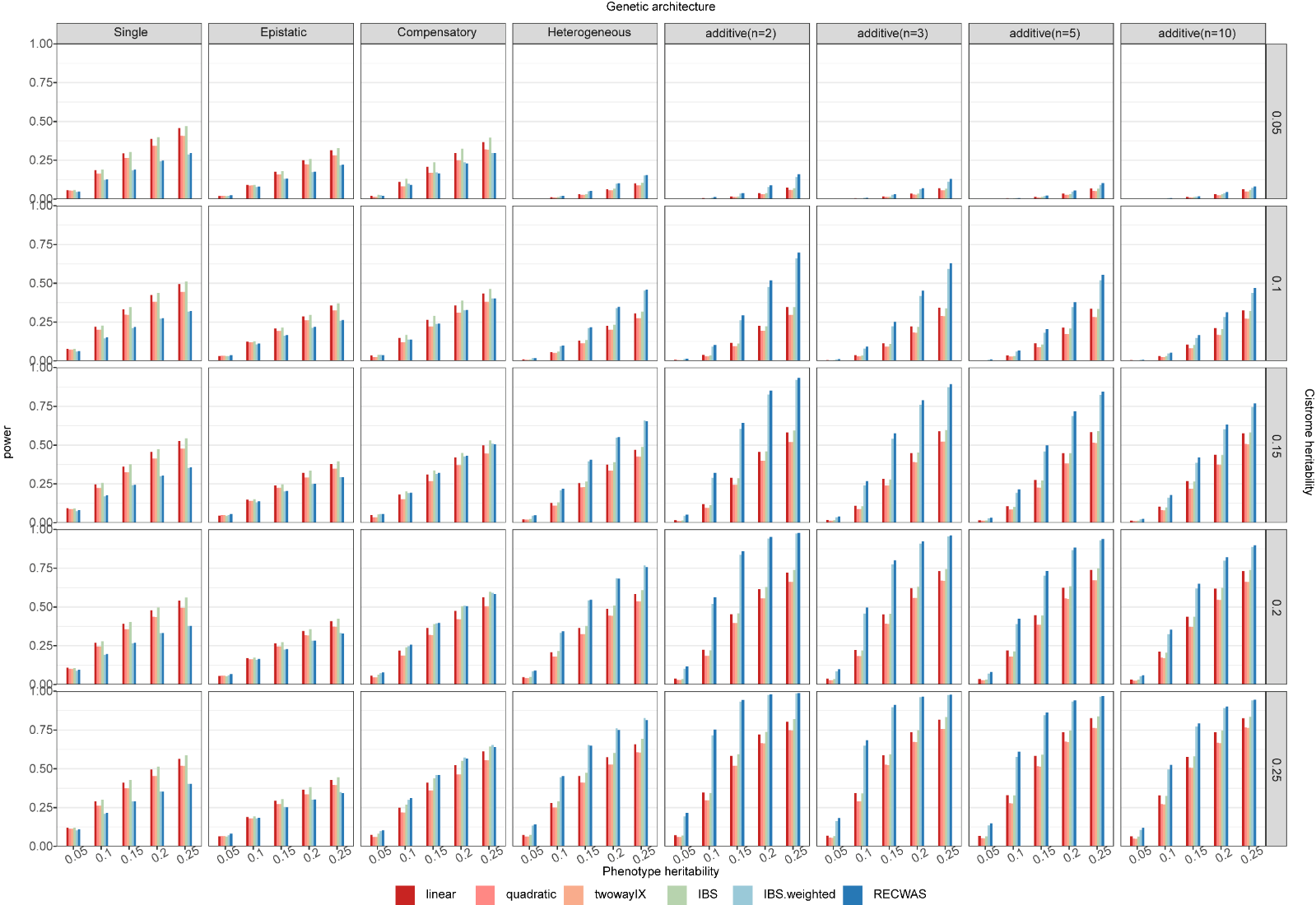


Figure S3. Statistical power (left-y-axis) of different kernels compared on causality model simulated at varying levels of cistrome heritability (right-y-axis), phenotype heritability (x-axis), and different genetic architectures including single, epistatic, compensatory, heterogeneous, and additive.


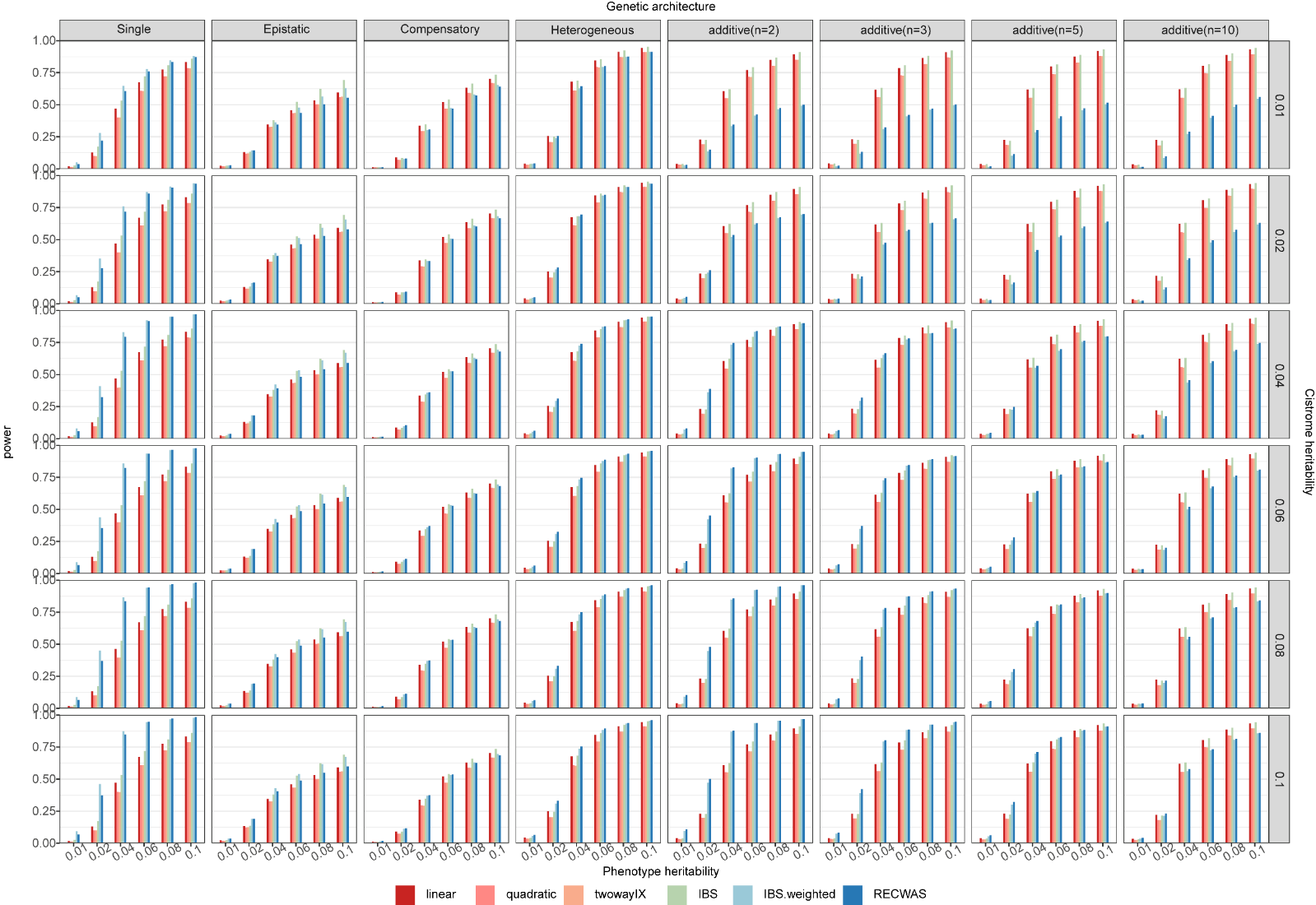


Figure S4. Statistical power (left-y-axis) of different kernels compared on pleiotropy model simulated at varying levels of cistrome heritability (right-y-axis), phenotype heritability (x-axis), and different genetic architectures including single, epistatic, compensatory, heterogeneous, and additive.


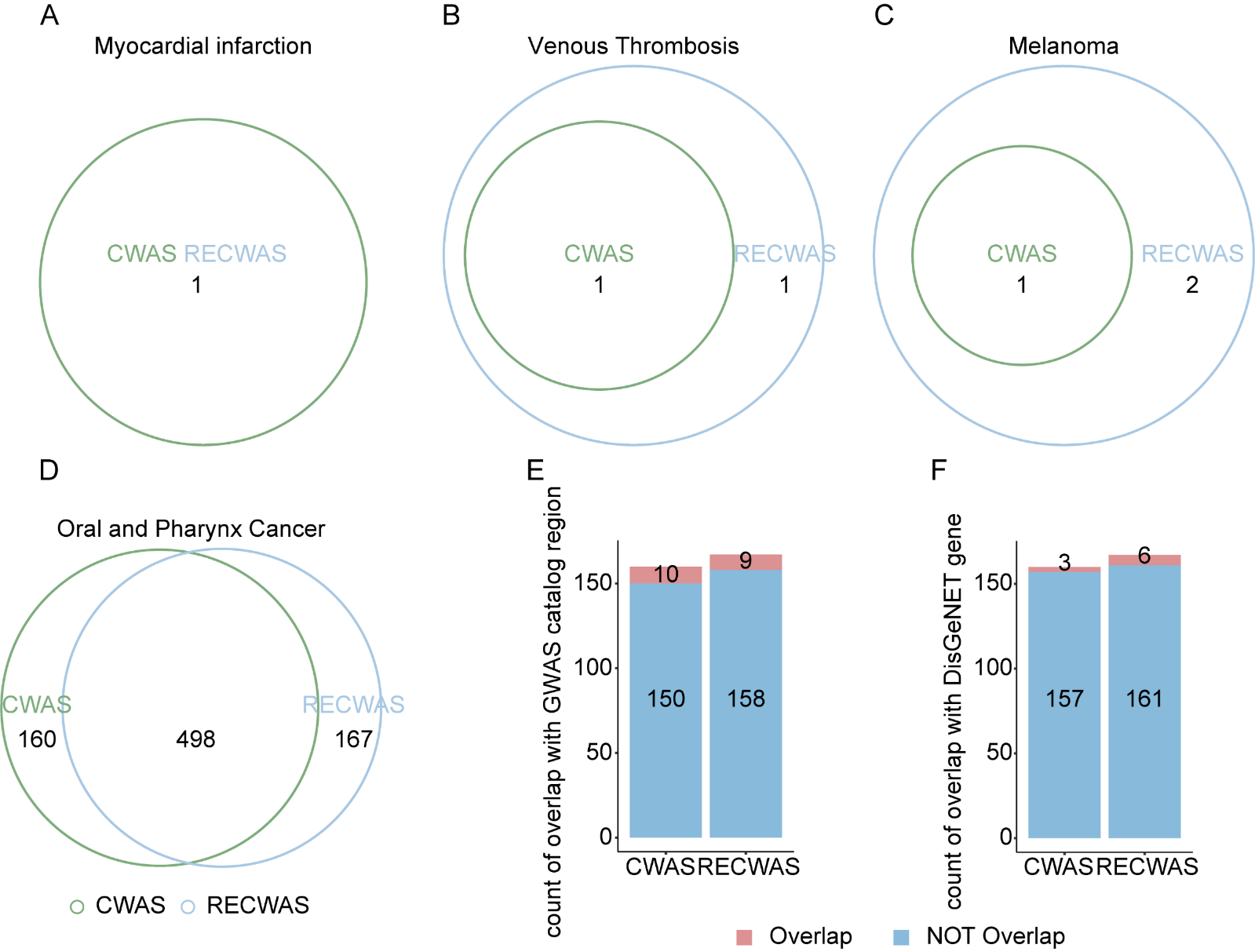


Figure S5. Overview of significant peaks identified by CWAS and RECWAS in non-prostate cancer data. Overlap of significant peaks identified by CWAS and RECWAS in (A) myocardial infarction, (B) venous thrombosis, (C) melanoma, and (D) oral and pharynx cancer. (E) The number of significant peaks located in the oral and pharynx cancer GWAS risk region, extracted from the GWAS catalog, uniquely identified by CWAS and RECWAS. (F) The number of significant peaks located in oral and pharynx cancer susceptibility regions, extracted from DisGeNET, uniquely identified by CWAS and RECWAS.


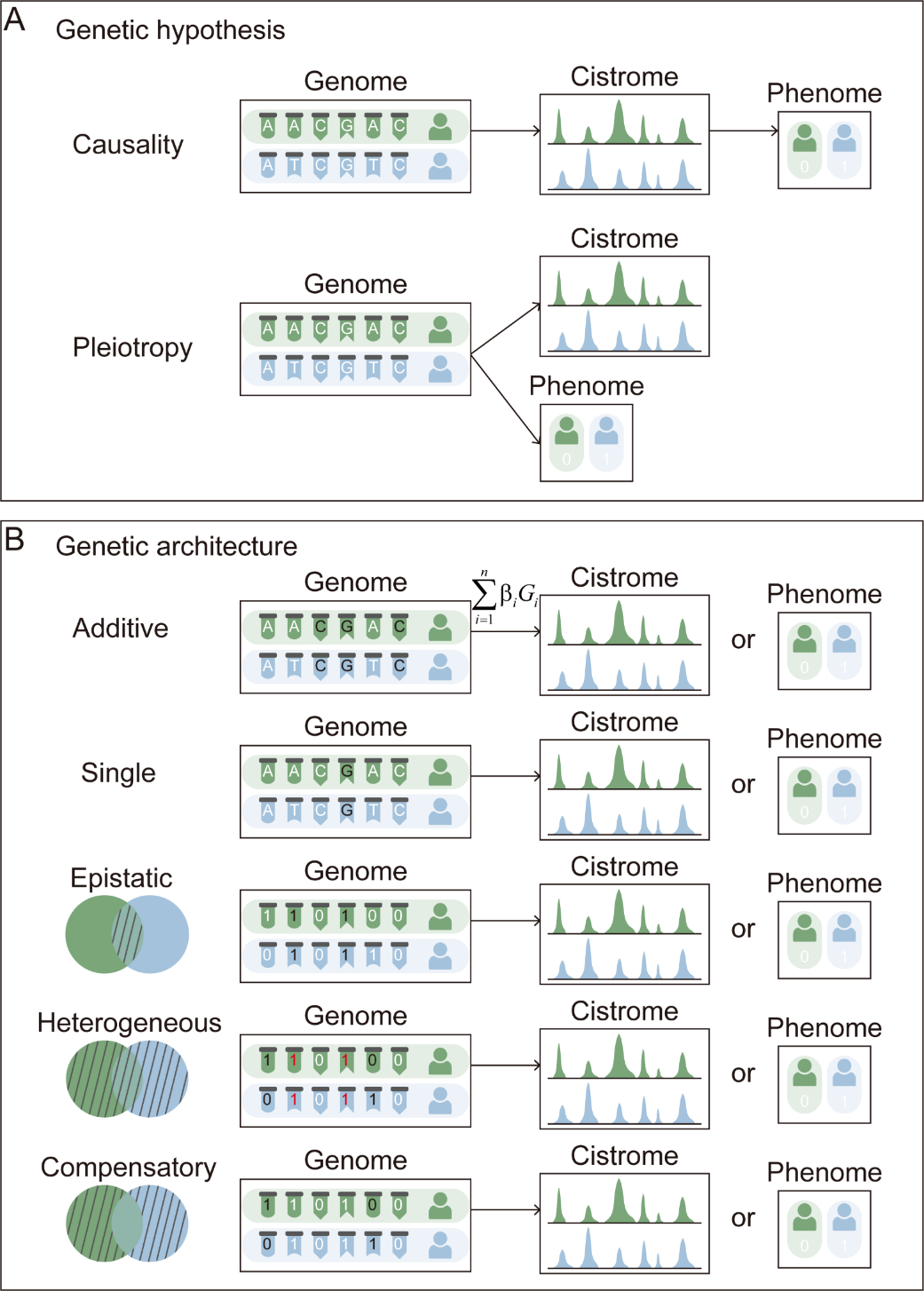


Figure S6. Detailed illustration of different genetic scenarios. (A) Genetic hypothesis: causality and pleiotropy. Causality: genome affects phenome via cistrome. Pleiotropy: genome affects cistrome and phenome simultaneously. (B) Genetic architecture: additive, single, epistatic, heterogeneous, compensatory. Additive: joint effect of multiple SNPs in a linear manner to cistrome and phenome. Single: effect of one SNP on cistrome or phenome. Epistatic: two SNPs with mutated alleles affect cistrome or phenome. Heterogeneous: one or two SNPs with mutated alleles can both affect cistrome or phenome. Compensatory: one of two SNPs with a mutated allele affects cistrome or phenome.
